# Alloying Ag_2_S Quantum Dots with Gold: Controlling NIR‐I Emission with Material Modification

**DOI:** 10.1002/smll.202505308

**Published:** 2025-09-01

**Authors:** Kanik Chelani, Thomas W. Price, Victor Gonçalves, Ihsan Çaha, Francis Leonard Deepak, Rafael T. M. de Rosales, Juan Gallo, Graeme J. Stasiuk

**Affiliations:** ^1^ Department of Imaging Chemistry and Biology School of Biomedical Engineering and Imaging Sciences St Thomas' Hospital King's College London 4th Floor Lambeth Wing London SE1 7EH UK; ^2^ Advanced Magnetic Theranostic Nanostructures Laboratory International Iberian Nanotechnology Laboratory Av. Mestre José Veiga Braga 4715‐330 Portugal; ^3^ Nanostructured Materials Research Group International Iberian Nanotechnology Laboratory Av. Mestre José Veiga Braga 4715‐330 Portugal

**Keywords:** alloying, fluorescence, gold, near‐infrared, quantum dots, silver, tunable

## Abstract

Herein, the preparation of tunable NIR‐I emitting (700–900 nm) Au‐alloyed Ag_2_S QDs is outlined, controlled through Au:Ag reaction stoichiometry. Increasing Au:Ag results in blue‐shifted emission (*λ*_em_ = 1180–715 nm), accompanied with an increased measured bandgap (E_g_ = 1.6–2.3 eV). Au‐alloyed Ag_2_S QDs exhibited enhanced PLQYs (φ = 26.7%) and prolonged lifetimes (τ = 4.57 µs) compared to their parent Ag_2_S QDs (φ = 5.9%, τ = 1.87 µs). Detailed structural characterization revealed that the series of NIR QDs possessed uniform size distributions with morphological changes due to the nucleation of Au on the QD surface. Cationic exchange of Ag^+^ for Au^+^ occurs, originating from these nucleation sites. Phase transformations in the crystal structure were evident, giving rise to the evolution of Ag_3_AuS_2_, AgAuS, and AgAu_3_ species. AgAuS QDs were transferred to aqueous media through ligand exchange with dihydrolipoic acid (DHLA), to produce colloidally stable QDs with an emission maximum in the far NIR‐I region at 845 nm. NIR‐I imaging phantoms were additionally measured to confirm their compatibility with commercial instrumentation. To our knowledge, this is the first report elucidating the relationship between structural evolution and optoelectronic properties of tunable NIR‐I emitting Au‐alloyed Ag_2_S QDs.

## Graphical abstract

Summarised physical and optical properties of Au‐alloyed Ag_2_S QDs using 0–3 eq. HAuCl_4_ with respect to Ag in the reaction. Emission spectra (centre) and HAADF‐STEM images of QD structures containing Ag_2_S(bottom‐right), Ag_3_AuS_2_ (top‐right), AgAuS (top‐left), and AgAu_3_‐AgAuS (bottom‐left) respectively.

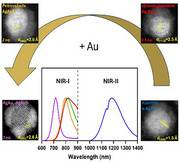

## 1 Introduction

Quantum dots (QDs) are nanoparticles composed of semiconducting materials that exhibit quantum confinement effects, resulting in distinctive photophysical properties.^[^ ^1^ ^]^ QDs can emit across the electromagnetic spectrum, from the visible (380–700 nm) to the near‐infrared (NIR) region (700–1600 nm). The optical properties of QDs are dependent on their composition and size.^[^ ^2^ ^]^ Compared to organic dyes, QDs offer advantages such as greater resistance to photobleaching, larger Stokes shifts, narrower emission spectra, and relatively high photoluminescent quantum yields (PLQYs).^[^ ^3^ ^]^ Moreover, the surface chemistry of QDs may be exploited to incorporate a range of ligands for diverse applications.^[^ ^4^ ^]^ QDs have been widely used in displays, photovoltaics, and LEDs, as well as in electronic applications like quantum computing, transistors, and solar cells due to their quantum confinement effects.^[^ ^5^, ^6^, ^7^, ^8^ ^]^ Their use in biomedicine is also rapidly growing and has been extensively studied over the past two decades. The reduced scattering and absorption of biological tissues in the near‐infrared (NIR) region call for the development of NIR‐emitting QDs for deep‐tissue bioimaging applications.^[^ ^9^ ^]^ Furthermore, autofluorescence from tissues is also reduced in the NIR range; therefore, a greater fluorescence signal‐to‐noise ratio may be achieved by imaging in the NIR range.

In the last decade, Ag‐based QDs have emerged as promising NIR emitting agents for biomedical imaging applications due to their inherently low in vivo cytotoxicity relative to QDs composed of more traditional heavy metals such as Cd or Pb.^[^ ^10^, ^11^, ^12^ ^]^ Ag‐based QDs have typically been reported to possess tunable emission profiles within the NIR‐II region (1000–1600 nm).^[^ ^13^, ^14^, ^15^ ^]^ Although NIR‐II imaging holds potential for diverse applications, such as preclinical studies in research, clinical adoption remains at an early stage.^[^ ^16^ ^]^ Key challenges, including device size reduction, cost‐effectiveness, regulatory approval, and validation, must be overcome before NIR‐II imaging systems can be utilised extensively in clinical settings. Therefore NIR‐I emitters may be more compatible since biomedical devices, such as handheld NIR‐I (700–900 nm) imaging systems, are readily employed together with NIR‐I dyes (e.g. indocyanine green, λ_em_ = 813 nm) for intraoperative imaging applications.^[^ ^17^, ^18^ ^]^

**Figure 1:**
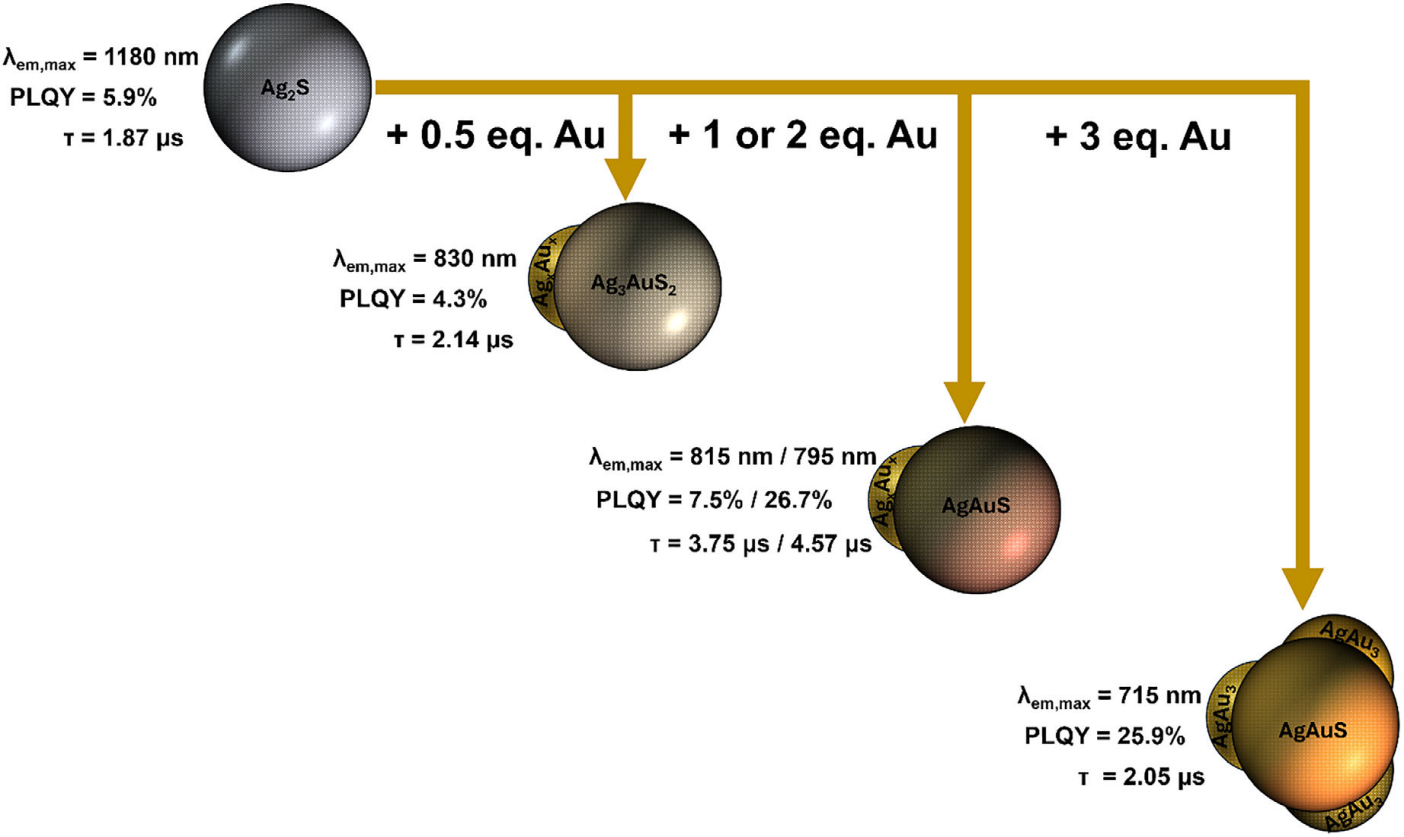
Scheme showing the effect of alloying Ag_2_S QDs with Au; control of HAuCl_4_ equivalents in the synthesis yields different morphologies, structures, and enables tunability of optical properties.

Recently, Yang et al. reported the synthesis of NIR emitting Ag_2_Se QDs followed by an Au‐alloying step *via* cationic exchange processes to produce AgAuSe QDs.^[^ ^19^ ^]^ Dalmases et al. have also previously studied cationic exchange of Au onto larger Ag_2_S nanoparticles (*d* = 10–20 nm), elucidating structural/morphological changes. Following from these works, we have developed a novel synthetic procedure utilising NIR‐II emitting Ag_2_S QDs (λ_em_ = 1180 nm) to produce a range of NIR‐I emitting AgAuS QDs (λ_em_ = 715–830 nm) *via* Au‐alloying methodology, as shown in the scheme below (**Figure** **1**). Furthermore, we have been able to correlate these photophysical changes to the physical transformations induced by the alloying process. To further advance this new technology toward imaging applications, we have demonstrated a phase transfer process to produce water‐dispersible AgAuS QDs, retaining their optimised optical properties.

## 2 Results and Discussion

### 2.1 Synthesis of Ag_2_S QDs

**Figure 2:**
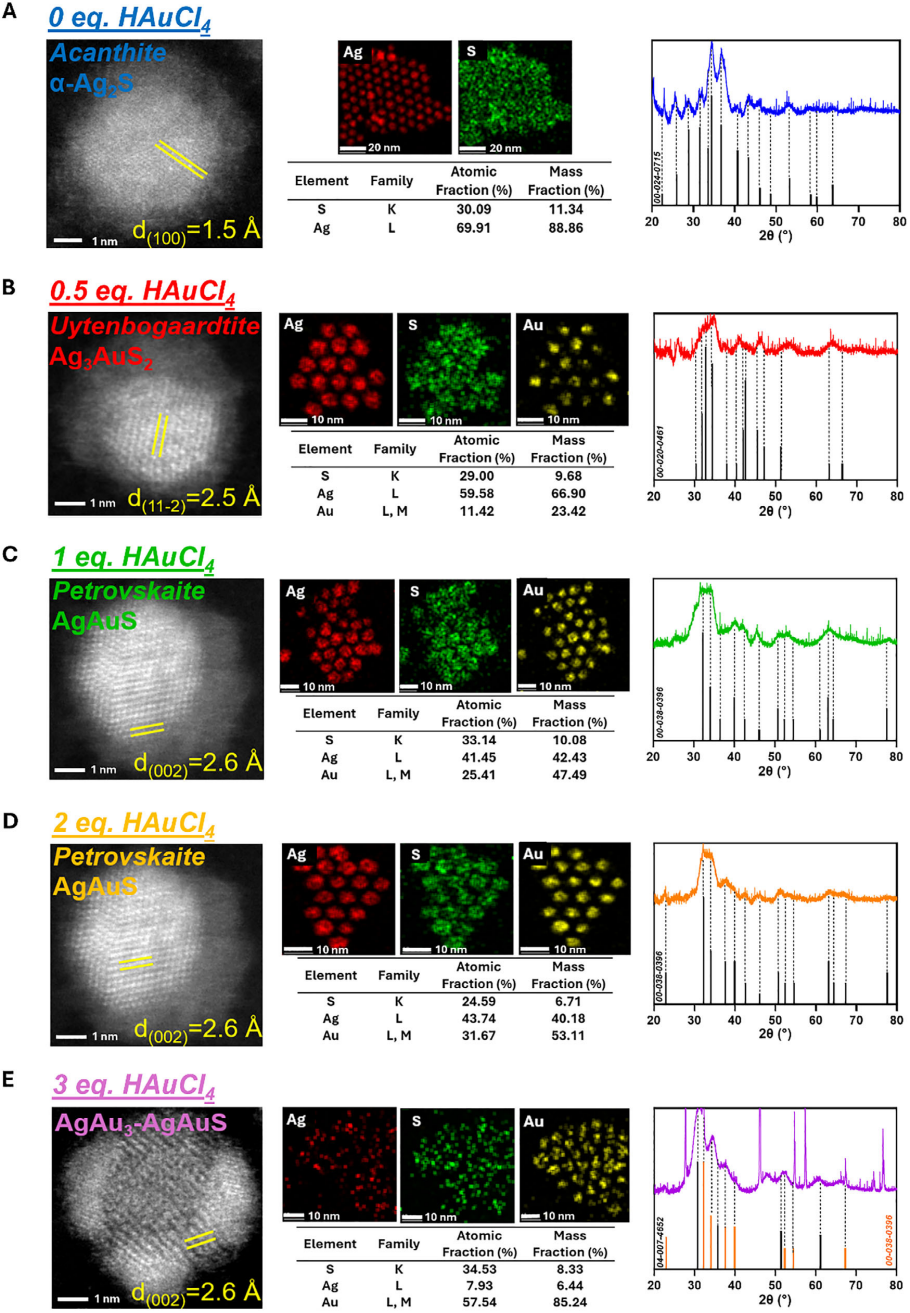
HAADF‐STEM (left), EDX map with corresponding tabulated data (middle), and reference matched XRD spectra (right) of A) Ag_2_S QDs, alloyed with B) 0.5 eq., C) 1 eq., D) 2 eq., and E) 3 eq. HAuCl_4_.

**Figure 3:**
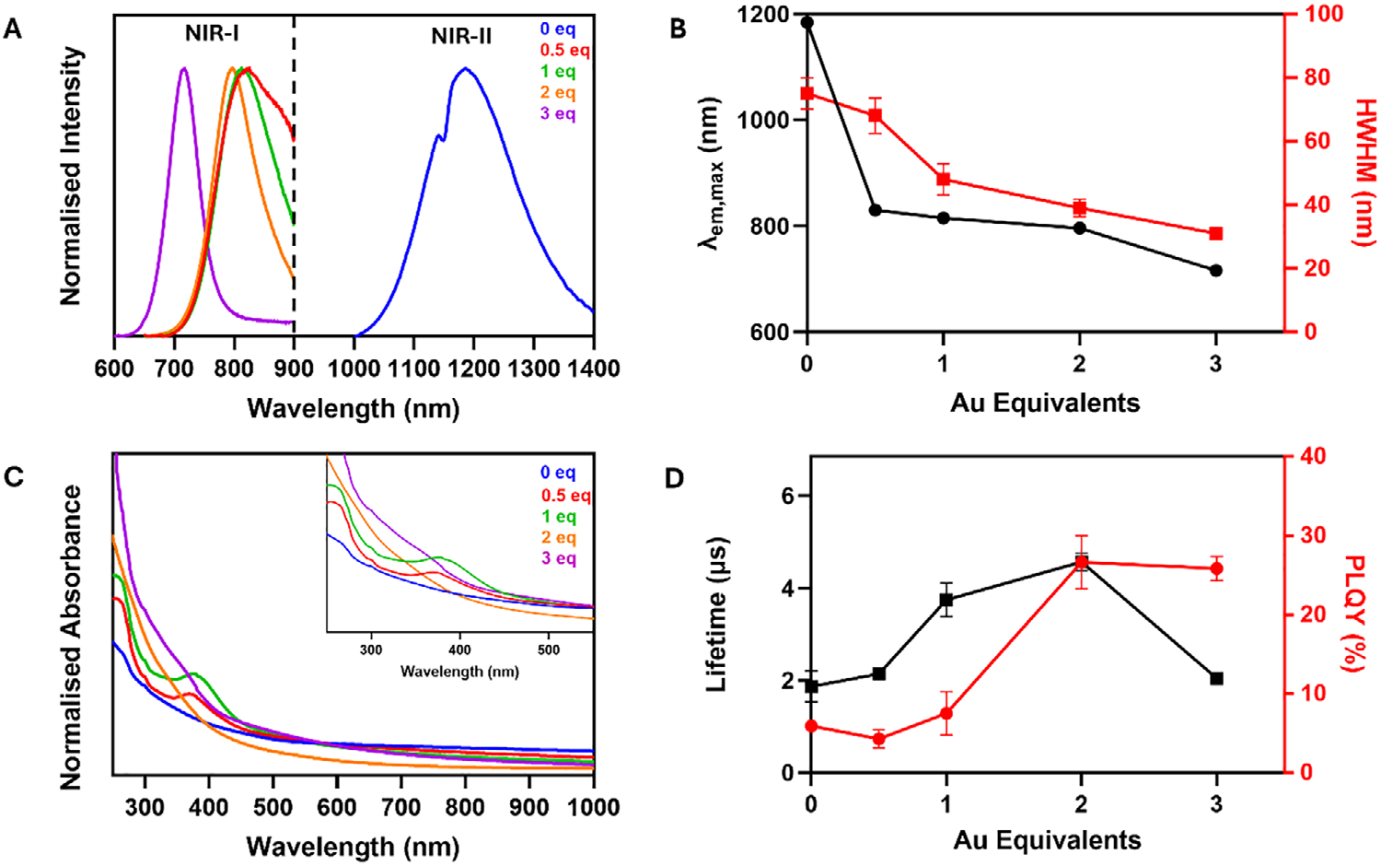
A) Normalised emission spectra of Ag_2_S QDs, (λ_ex_ = 400 nm), Au‐alloyed Ag_2_S QDs using 0.5 eq. (λ_ex_ = 395 nm), 1 eq. (λ_ex_ = 395 nm), 2 eq. (λ_ex_ = 395 nm), and 3 eq. (λ_ex_ = 415 nm) of Au with respect to Ag. B) Corresponding emission maxima and half‐widths at half‐maximum (HWHM) plotted against the number of equivalents of Au in the reaction. C) Normalised absorption spectra of Au‐alloyed Ag_2_S QDs. D) Measured fluorescence lifetimes and PLQYs of corresponding QDs plotted against Au equivalents in the reaction. All spectra were measured in chloroform at 25 °C. Original spectra may be found in the ESI.

The synthesis of Ag_2_S QDs was adapted from Zhang et al. by modifying the nanocrystal growth time.^[^ ^20^ ^]^ These QDs possess a spherical morphology with a narrow size distribution centered at an average diameter of *d* = 6.38 ± 0.52 nm (**Figure** **2**A; Figure S1A–D, Supporting Information).^[^ ^20^ ^]^ The crystal structure was determined to be a monoclinic system (*P2_1_/n*), consistent with *Acanthite* Ag_2_S. The fast‐Fourier transform (FFT) of a QD in HAADF‐STEM image confirmed that the Ag_2_S was oriented along the (111) zone axis and a d‐spacing of 1.5 Å for the (100) plane was measured (Figure S1, Supporting Information). This structure was confirmed through XRD and is in good agreement with a standard reference for monoclinic Ag_2_S (JCPDS 00‐024‐0715).^[^ ^21^ ^]^ Elemental mapping indicates a uniform distribution of sulfur through the nanoparticles, with Ag following a similar pattern. This is reflected in the atomic ratios determined through EDX (Ag:S, 1:2.33), very close to the ideal 1:2 Ag_2_S ratio. The XPS data likewise confirms the presence of the expected peaks from Ag 3d (3d_5/2_ = 367.84 eV, 3d_3/2_ = 373.82 eV) and S 2p (2p_3/2_ = 161.55 eV, 2p_1/2_ = 162.85 eV) orbitals (Figure S2, Supporting Information). These peaks from Ag 3d_5/2_ and S 2p_3/2_ are consistent with reported ranges for Ag^+^ and S^2−^, and align with previous syntheses of Ag_2_S QDs.^[^ ^22^ ^]^ These QDs possessed a broad emission, centered at 1180 nm (**Figure** **3**A; Figure S3A, Supporting Information). PLQY and emission lifetime, measured to be 5.9 ± 0.7% and 1.87 ± 0.34 µs respectively, are also in line with those reported for Ag_2_S QDs produced *via* similar procedures (Figure S3B,C and Table S1, Supporting Information).^[^ ^9^, ^20^ ^]^ Ag_2_S QDs synthesised in this work were estimated to possess an E_g_ of 1.60 eV, not too dissimilar to the literature value of 1.54 eV for similarly sized QDs, as demonstrated by Sadovnikov et al. (Figure S4A, Supporting Information).^[^ ^23^ ^]^

### 2.2 Au‐alloyed Ag_2_S QDs

The cationic exchange Au‐alloying procedure was then applied to the synthesised Ag_2_S QDs to produce a series of Ag_x_Au_x_S_x_ QDs through stoichiometric control.^[^ ^19^ ^]^ Ag_2_S QDs were alloyed with Au by incubation with HAuCl_4_ in chloroform overnight, followed by precipitation to remove excess salts. A series of samples were prepared by varying the amount of Au used in the reaction relative to the Ag concentration; herein referred to by the number of equivalents of Au added to the reaction.

The alloying produced QDs with striking differences in their emission properties; upon increasing the number of Au equivalents (0.5–3 eq.), a blueshift in emission (from *λ*_em_ = 1180 nm to 715–830 nm) was observed (Figure 3A,B). This brought the QD emission from the NIR‐II region to within the NIR‐I region. As the Au content was increased, the emission spectra also narrowed, as shown by the reduction in the respective half‐widths at half‐maxima (HWHM), from 75 nm for Ag_2_S QDs, to 31 nm for AgAuS with 3 eq. Au. Furthermore, an increase in the PLQY was observed in all cases except for the 0.5 Au equivalents. These changing properties prompted further investigation to verify the underlying mechanisms of physical and electronic transformations upon alloying of Ag_2_S with HAuCl_4_. The original Ag_2_S absorption spectrum is consistent with most Ag‐based QDs that typically do not display any visible excitonic peak (Figure 3C).^[^ ^20^ ^]^ Notably, the QDs alloyed with 0.5 and 1 eq Au displayed an additional peak at 400 nm. The absorption band near 400 nm is reminiscent of the SPR of metallic Ag; however, in the absence of direct evidence for Ag⁰ formation, this feature may be attributed to electronic transitions associated with intermediate Ag^+^ based species or lattice modifications.^[^ ^24^ ^]^ Tauc plots were produced from this data to estimate the semi‐conductor bandgap energy (E_g_) (Figure S4, Supporting Information).^[^ ^25^ ^]^ The physical properties of these Au‐alloyed Ag_2_S QDs were characterised in detail to elucidate their size, morphology, elemental composition, and crystal structure to correlate structure and optical properties.

#### 2.2.1 0.5 eq. Au‐alloyed Ag_2_S QDs

Ag_2_S alloyed with 0.5 eq. of HAuCl_4_ produced a slight reduction in diameter to 6.04 ± 0.63 nm. (Figure 2B; Figures S5A–D and S23, Supporting Information), suggesting etching upon nucleation of Au onto the surface of the QDs.^[^ ^19^, ^26^ ^]^ A change in phase to a ditrigonal pyramidal crystal system (*R3c*) consistent with *Uytenbogaardtite* Ag_3_AuS_2_ was observed (JCPDS 00‐020‐0461). These Ag_3_AuS_2_ nanocrystals possess a d‐spacing of 2.5 Å for the (121) plane, measured along the (111) zone axis (Figure S5, Supporting Information). The likely explanation behind this increase is due to the larger ionic radii of Au^+^ (151 pm) than the cationic exchanged Ag^+^ (129 pm).^[^ ^27^ ^]^ Regions of the nanoparticle that exhibit brighter contrast in HAADF‐STEM images are proposed to be the nucleation sites of Au during cationic exchange, resulting in the formation of Janus‐like QDs.^[^ ^28^, ^29^ ^]^ This nucleation process is challenging to address, even with the use of surfactants. Some reports have linked this process to the availability of agents for the reduction of Au^3+^ to Au^+^.^[^ ^30^ ^]^ Further evidence of alloying is observed through the observation of Au signals in XPS and EDX measurements. A 2.85:1 Ag:Au ratio was observed through EDX, in good agreement with the Ag_3_AuS_2_ structure (Figure 2B). Elemental mapping demonstrates that Ag and Au are distributed throughout the nanomaterial. This is consistent with the cationic exchange mechanism due to the Kirkendall effect, as there is a high affinity for Au^+^ to Ag^+^ chalcogenide lattices.^[^ ^31^ ^]^ Ultimately, this results in the formation of highly stable ternary QDs, with a kinetically favourable transformation that occurs rapidly at room temperature. The XPS spectrum of these QDs displayed characteristic peaks at different binding energies corresponding to Ag 3d, Au 4f, and S 2p orbitals (Figure S6, Supporting Information). Ag 3d_5/2_ and 3d_3/2_ gave binding energies of 367.54 and 373.53 eV respectively, negatively shifted from Ag_2_S QDs by 0.3 eV. Likewise, S 2p_3/2_ and 2p_1/2_ at 160.97 and 162.08 eV were reduced by 0.6 and 0.8 eV respectively. This data suggests that the Ag_3_AuS_2_ phase possesses less bound photoelectrons than Ag_2_S. The change in material is also evidenced through the increase in bandgap energy to 1.95 eV, and a concomitant 350 nm blueshift of the emission maxima from 1180 to 830 nm (Figure 3A; Figure S4B, Supporting Information). These differences in the measured E_g_ are consistent with the observed blueshifts in the emission spectra upon increasing the Au content. According to multiple reports, Au incorporation alters the position of the conduction band, while retaining similar valence band energy, thereby changing E_g_.^[^ ^19^, ^32^ ^]^ The PLQY of QDs alloyed with 0.5 eq. Au was measured to be 4.3 ± 1.2%, very close to the original from Ag_2_S QDs (Figure S7, Supporting Information). However, the emission lifetime increased slightly to 2.14 ± 0.06 µs (Table S2, Supporting Information).

#### 2.2.2 1 eq. Au‐alloyed Ag_2_S QDs

**Figure 4:**
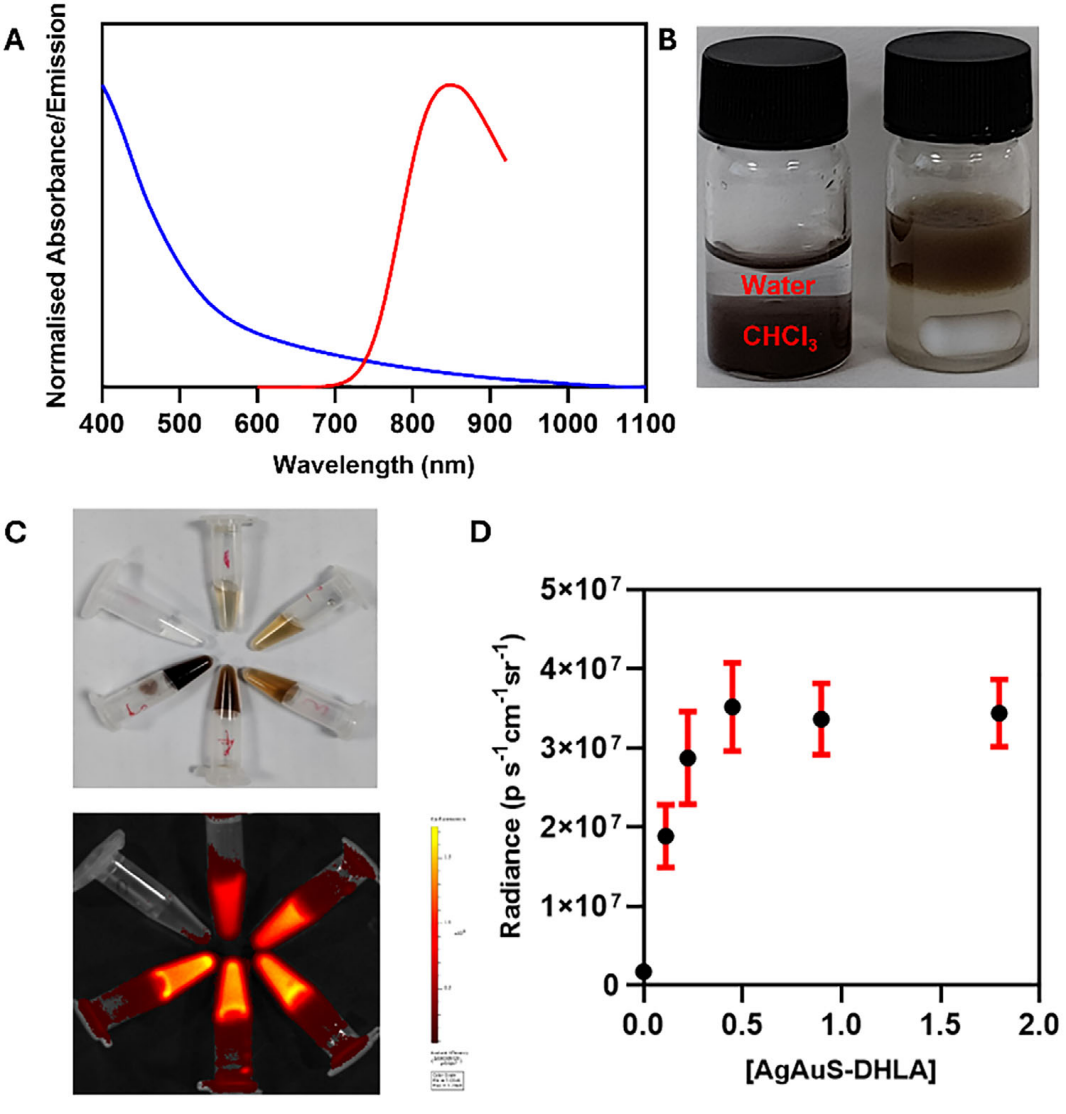
A) Absorption (blue) and emission (red) spectra (λ_ex_ = 395 nm) of AgAuS QDs phase transferred with DHLA. B) Photograph of a biphasic reaction mixture to show transfer of dark brown AgAuS QDs from the lower chloroform to the upper water layer. C) Photographs of serially diluted QDs under white light (top), NIR‐I fluorescence image of corresponding QDs measured using a bandpass filter of 840 nm (λ_ex_ = 410 nm). D) Average radiance obtained by measuring ROIs from NIR‐I fluorescence images plotted against AgAuS‐LA QD concentration.

Alloying with 1 eq. HAuCl_4_ reduced further the average diameter (*d* = 5.66 ± 0.64 nm) while retaining the Janus‐like appearance (Figure 2C; Figures S8A–D and S24, Supporting Information). The formation of *Petrovskaite* AgAuS in a monoclinic crystal system (*P2/m)* was observed, consistent with the standard reference (JCPDS 00‐038‐0396). However, EDX analysis provided an overall Ag:Au ratio of 1.63:1, supporting the observation of regions of AgAuS on the surface of an Ag_2_S or Ag_3_AuS_2_ core. These regions were aligned along the (110) zone axis, and the lattice spacing for the (002) plane increased slightly up to 2.6 Å, which may be dependent on the concentration of the alloying species (Figure S8, Supporting Information).^[^ ^33^ ^]^ XPS showed a negligible difference from the 0.5 eq. QDs in Ag 3d_5/2_ and 3d_3/2_ peaks at 367.56 and 373.55 eV (Figure S9, Supporting Information). Likewise, Au 4f_5/2_ and 4f_7/2_ peaks at 84.12 and 87.79 eV were at identical binding energies. S 2p_3/2_ and 2p_1/2_ however, shifted positively by 0.10 eV to 161.06 and 162.17 eV, respectively, suggesting increased binding interactions of S^2−^ with Au^+^ and Ag^+^. The band gap energy increased further to 2.25 eV, with a further blue shift in emission to 815 nm (**Figures** 3A, **4**C). PLQY and emission lifetimes both increased to 7.5 ± 2.7% and 3.75 ± 0.37 µs, respectively (Figure S10 and Table S3, Supporting Information). These photoluminescent enhancement effects are proposed to be due to the filling of cationic vacancies and defects in the crystal lattice by the incorporation of Au^+^.^[^ ^19^ ^]^ This would ultimately result in a lower probability for non‐radiative excitonic transitions; hence, a greater proportion of excitons are radiative.^[^ ^34^, ^35^ ^]^

#### 2.2.3 2 eq. Au‐alloyed Ag_2_S QDs

Upon alloying with 2 eq. of HAuCl_4_, structural features similar to the 1 eq. synthesis was observed. The diameter remained unchanged (*d* = 5.76 ± 0.61 nm), and the crystal structures observed in these nanocrystals were identical (Figure 2D; Figures S11A–D and S25, Supporting Information). The Ag:Au ratio decreased to 1.38:1 suggesting that the ratio of AgAuS:Ag‐rich structure shifted slightly. Ag 3d_5/2_ and 3d_3/2_ photoelectric peaks at binding energies of 367.70 and 373.70 eV were observed, a 0.15 eV positive shift from the 1 eq. synthesis (Figure S12, Supporting Information). Au 4f_5/2_ and 4f_7/2_ peaks at 84.25 and 87.93 eV gave a similar positive shift of 0.14 eV. S 2p_3/2_ and 2p_1/2_ showed a more significant increase of up to 0.6 eV to 161.54 and 162.77 eV respectively. Hence, these alloyed QDs possess the most bound photoelectrons comparatively. A blueshift in emission to 795 nm was observed, although no change in E_g_ from the 1 eq. synthesis was recorded (Figure 3A; Figure S4D, Supporting Information). Both the PLQY and emission lifetimes of this sample were significantly higher at 26.7 ± 3.4% and 4.57 ± 0.19 µs respectively (Figure S13 and Table S4, Supporting Information). This indicates a further reduction in trap states likely due to greater filling of cationic vacancies in the crystal lattice.^[^ ^30^ ^]^

#### 2.2.4 3 eq. Au‐alloyed Ag_2_S QDs

Upon the addition of 3 eq. of HAuCl_4_, the morphology changed from Janus to core nanoparticles with multiple islands of Au nucleation (Figure 2E; Figures S14A–D and S26, Supporting Information).^[^ ^28^, ^29^, ^36^ ^]^ The diameter remained in a similar range (*d* = 5.67 ± 0.60 nm); however, clear changes to the crystal structure were observed. These nanostructures were composed of AgAuS cores decorated with AgAu_3_ islands. The multiple nucleation sites of Au enable cationic exchange to occur more rapidly at room temperature. Hence, the Ag:Au ratio decreased significantly to 0.14:1 to support these structural observations. AgAu_3_ with a cubic crystal system (*Pm*‐*3m*) on the surface of monoclinic AgAuS (*P2/m*) was observed. These AgAu_3_ regions were oriented along the (100) zone axis, in line with cubic Au_2_S (JCPDS 04‐007‐4652).^[^ ^30^, ^37^, ^38^ ^]^ The XRD spectra also exhibited sharp peaks that match AgCl, as a large amount of Ag^+^ ions were ejected from the lattice, and these would combine with Cl^−^ ions from the gold precursor to form this species (Figure S14F, Supporting Information).^[^ ^19^, ^39^ ^]^ This provides further evidence of the proposed cationic exchange mechanism for the outlined transformations. The loss of affinity of both Ag^+^ and Au^+^ was evidenced by the reduction in their binding energies (Figure S15, Supporting Information). Ag 3d peaks were observed at 367.26 and 373.23 eV corresponding to 3d_5/2_ and 3d_3/2_, which suffered a negative shift of up to 0.47 eV. Au 4f peaks were observed at 84.06 and 87.73 eV, related to 4f_5/2_ and 4f_7/2_; their binding energies also decreased by 0.20 eV, consistent with the ejection of Ag^+^ from the crystal lattice. Conversely, the S 2p peaks corresponding to 2p_3/2_ and 2p_1/2_ increased in their binding energies to 162.34 and 163.54 eV, respectively. This data originates from the stronger binding interactions of S^2−^ with Au^+^ than Ag^+^ in the primarily cubic AgAu_3_ regions of these QDs. The measured band gap increased to 2.30 eV to explain the blueshift to 715 nm (Figure 3A; Figure S4E, Supporting Information). The PLQY remained in a similar range at 25.9 ± 1.6%, while the emission lifetime reduced by more than half to 2.05 ± 0.13 µs (Figure S16 and Table S5, Supporting Information).

To summarise, morphological changes were observed post‐incorporation of Au (**Table** **1**). Upon addition of 0.5–2 eq. of HAuCl_4_, the spherical Ag_2_S QDs changed to Janus‐type structures.^[^ ^33^, ^34^ ^]^ This was accompanied by an evolution in the crystalline structure from Ag_2_S to Ag_3_AuS_2_ (0.5 eq.), and AgAuS (1–2 eq.).

**Table 1:** Summary of optical and physical characterisation of synthesised Au‐alloyed Ag_2_S QDs (0.5–3 eq. Au). All optical characterisation measurements were made in chloroform at 25 °C.

Au eq.	λ_ex_ [nm]	λ_em, max_ [nm]	PLQY [%]	τ [µs]	Eg [eV]	d [nm]	Compound	Crystal system
0	400	1180	5.9 ± 0.7	1.87 ± 0.34	1.60	6.38 ± 0.52	Ag_2_S *Acanthite*	Monoclinic (*P2_1_/n*)
0.5	395	830	4.3 ± 1.2	2.14 ± 0.06	1.95	6.04 ± 0.63	Ag_3_AuS_2_ *Uytenbogaardtite*	Ditrigonal pyramidal (*R3c*)
1	395	815	7.5 ± 2.7	3.75 ± 0.37	2.25	5.66 ± 0.64	AgAuS *Petrovskaite*	Monoclinic (*P2/m*)
2	395	795	26.7 ± 3.4	4.57 ± 0.19	2.25	5.76 ± 0.61	AgAuS *Petrovskaite*	Monoclinic (*P2/m*)
3	415	715	25.9 ± 1.6	2.05 ± 0.13	2.30	5.67 ± 0.60	AgAu_3_‐AgAuS *Petrovskaite*	Monoclinic (*P2/m*)

These alloyed structures displayed an increased bandgap from 1.60 to 1.95 eV, then to 2.25 eV. A concomitant blueshift in the emission maxima from 1180 to 830 nm, then to 795 nm was observed to demonstrate NIR‐I region tunability. However, the non‐linear nature of this blueshift suggests a structural rather than stoichiometric mechanism for this change in emission.^[^ ^40^, ^41^ ^]^ Further increasing the added Au to 3 eq. resulted in core AgAuS nanomaterials, with the formation of AgAu_3_ islands on the bulk nanocrystals.^[^ ^36^ ^]^ This material has a bandgap of 2.30 eV and an emission at 715 nm. Moreover, these transformations also enhanced photoluminescent properties such as PLQY and lifetimes. This was due to the filling of cationic vacancies and defects in the crystalline lattice with Au^+^, resulting in reduced probability for non‐radiative recombination.^[^ ^19^, ^34^, ^35^ ^]^ Additionally, it is notable that the slight reduction in particle size, potentially due to etching of the surface, could likewise reduce surface defects and contribute to the observed photoluminescent enhancement effects.^[^ ^42^ ^]^ From the optical characterisation data, the 2 eq. Au synthesis yields optimised optical properties beneficial for imaging applications, while the long emission lifetimes could also have application in time‐resolved fluorescence imaging.^[^ ^43^ ^]^ Currently there are limited reports of analogous QD materials in the literature. Recently, only Yang et al. have reported the preparation of smaller‐sized AgAuS QDs (*d* = 2–3 nm, λ_em_ = 707 nm) using a different synthetic methodology.^[^ ^32^ ^]^ However, their procedure requires several days for synthesis and purification, while only yielding a very broad far‐red emission. The Au‐alloyed Ag_2_S QDs produced in our novel, relatively facile synthesis therefore offer the advantage of having a narrow, tunable emission within the central NIR‐I window, among other photoluminescent enhancement properties described above.

### 2.3 Phase Transfer of AgAuS QDs

The preparation of AgAuS QDs using 2 eq. of HAuCl_4_ yielded NIR‐I emitting QDs (λ_em_ = 795 nm) with an optimised PLQY and emission lifetime. Furthermore, these AgAuS QDs exhibit uniform composition and phase distribution, facilitating their potential for further optimisation and development. These AgAuS QDs were transferred from organic apolar solvents to the aqueous phase to study any changes to their emission profiles. A previously reported phase transfer protocol for Ag_2_S QDs was modified to consider reaction pH and the number of equivalents of the ligand dihydrolipoic acid (DHLA).^[^ ^20^ ^]^ Phase transfers were performed using an excess (400 000 eq.) of DHLA under weakly basic conditions (pH 7.6) to promote the replacement of the strongly bound, lipophilic capping agent 1‐dodecanethiol (DDT).^[^ ^44^ ^]^ The transfer of dark brown‐coloured AgAuS QDs from the lower chloroform layer to the upper aqueous layer in the biphasic reaction mixture was readily observed (Figure 4B). This preparation of hydrophilic AgAuS‐LA QDs offers a rapid and complete phase transfer at mild pH conditions in less than 30 min.

The hydrophilic AgAuS‐LA QDs were characterised through their absorption and emission spectra (Figure 4A). There was no observable change in the absorption spectra measured in water relative to that recorded in chloroform prior to phase transfer. AgAuS‐LA QDs possess an emission maximum at 845 nm, exhibiting a redshift of 50 nm from the emission spectra of QDs measured in chloroform (λ_em_ = 795 nm). This redshift may originate from the difference in electronic contributions from the sulfur coordination in LA compared to DDT. Giansante et al. proposed that the optical band gap is inversely proportional to the surface‐to‐volume ratio and that the occupied valence 3p electrons from sulfur‐containing ligands contribute to the valence band edge.^[^ ^45^ ^]^ This suggests an increased electronic contribution from the two coordinating thiols of LA compared to the single coordinating thiol from DDT, consistent with the observed redshift in emission spectra. The emission lifetime of these QDs was measured to be 1.40 ± 0.03 µs, which represents approximately a 3‐fold reduction from the lifetime measured in chloroform (Figure S19 and Table S6, Supporting Information). This reduction is a result of the well‐studied fluorescence quenching effect due to efficient overlap of O‐H bond vibrational energy levels of water (*ν* = 3, 1.46 eV) with the excited states of the QDs; hence, a greater probability for non‐radiative recombination is expected.^[^ ^46^ ^]^ AgAuS‐LA QDs possess a hydrodynamic diameter (D_H_) of 12.7 ± 2.7 nm and a zeta‐potential (ζ) value of ‐29.8 ± 1.8 mV (Figure S20, Supporting Information). The hydrodynamic diameter is greater than the observed nanoparticle diameter; this is expected due to the association of water molecules with the hydrophilic ligands containing negatively charged carboxylates. Similar D_H_ has been observed for analogous NPs with similar sizes and coatings.^[^ ^20^, ^44^ ^]^ The zeta‐potential value is evidence of this net negatively charged QD surface that provides sufficient electrostatic repulsion between nanoparticles to reduce aggregation and achieve colloidal stability.^[^ ^47^ ^]^

NIR‐I fluorescence phantoms were measured in serial dilutions of AgAuS‐LA QDs in water (0–2 µm) (Figure 4C). A bandpass filter of 840 nm was chosen for NIR imaging of these AgAuS‐LA QDs, not too dissimilar from their emission maxima at 845 nm. The lowest concentration tested, 100 nm, produced a strong signal, indicating this is well within the detection limits of the system. However, the average emission from the phantoms plateaued at higher concentrations (Figure 4D); the observed emission was far below the threshold of the instrument (10^10^ radiance units), indicating this concentration dependency is a property of the samples themselves. This effect may be the result of significant reabsorption of the emission at concentrations > 0.5 µm due to the high molar extinction coefficient (ε_400nm_) of 5.22 × 10^6^ m ^−1^ cm^−1^ measured for these QDs (Figure S21, Supporting Information).^[^ ^48^ ^]^

## 3 Conclusion

In this study, Ag_2_S QDs (λ_em_ = 1180 nm) were alloyed with different amounts of Au (0.5–3 eq.) to generate nanomaterials through a cationic exchange mechanism. These Ag_x_Au_x_S_x_ alloyed QDs displayed blue‐shifted emission spectra (λ_em_ = 715–830 nm) resulting from their increased band gap (E_g_ = 1.60–2.35 eV). Upon alloying Ag_2_S QDs with different amounts of Au, the spherical morphology evolved to Janus (0.5–2 eq.) and then patchy‐like (3 eq.) morphologies.^[^ ^28^, ^29^, ^36^ ^]^ These visible Au nucleation sites were consistent with proposed cationic exchange mechanisms.^[^ ^19^, ^30^, ^37^, ^38^ ^]^ Changes in crystal structures were observed as the incorporated Au content, confirmed by high‐resolution HAADF‐STEM, EDX, and XRD, was controlled through reaction stoichiometry. Three crystal structures were observed in Ag_3_AuS_2_ (0.5 eq.), AgAuS (1–3 eq.), and AgAu_3_ (3 eq.) that give rise to the changes in the measured E_g_ and the resulting emission spectra of the QDs. The synthesis of AgAuS QDs with the use of 2 eq. Au yielded the most bound photoelectrons, with an incorporated Ag:Au ratio measured at 1.38:1 through EDX and XPS. Moreover, these QDs possess the most ideal optical properties with a central NIR‐I emission (λ_em_ = 795 nm), PLQY of 26.7 ± 3.4% and a long emission lifetime of 4.57 ± 0.19 µs. AgAuS QDs were phase transferred into water *via* ligand exchange with DHLA to produce 50 nm redshifted emission spectra (λ_em_ = 845 nm). Furthermore, AgAuS‐LA QDs were characterised by their D_H_ and ζ‐potentials to demonstrate their inherent colloidal stability. The imaging potential of these water‐soluble QDs was proven through NIR‐I phantoms.

In conclusion, we have demonstrated the preparation of a range of novel Au‐alloyed Ag_2_S QDs with tunable optical properties in the NIR‐I region. This tunability was carried out through facile stoichiometric control of incorporated Ag:Au content, ultimately giving rise to differences in physical and optoelectronic properties.

## Conflict of Interest

The authors declare no conflict of interest.

## Supporting information



Supporting Information

## Data Availability

The data that support the findings of this study are available in the supplementary material of this article.
