# Supplementary material for: Alloying Ag2S Quantum Dots with Gold: Controlling NIR‐I Emission with Material Modification
Source: Small. 2025 Sep 1;21(41):e05308. doi: 10.1002/smll.202505308 (PMC12530014; doi:10.1002/smll.202505308)
Supplement: Supplementary file 1 — Supporting Information [file SMLL-21-e05308-s001.docx]

Supporting Information

Alloying Ag_2_S Quantum Dots with Gold: Controlling NIR-I Emission with Material Modification

Kanik Chelani,^a^ Thomas W. Price,^a^ Victor Gonçalves,^b^ Ihsan Çaha,^c^ Francis Leonard Deepak,^c^ Rafael T. M. de Rosales,^a^ Juan Gallo,^b^ and Graeme J. Stasiuk ^a^*

^a^ Department of Imaging Chemistry and Biology, School of Biomedical Engineering and Imaging Sciences, King's College London, 4th Floor Lambeth Wing, St Thomas' Hospital, SE1 7EH, London, UK.

^b^ Advanced Magnetic Theranostic Nanostructures Laboratory,

^c^ Nanostructured Materials Research Group, International Iberian Nanotechnology Laboratory, Av. Mestre José Veiga, 4715-330 Braga, Portugal

**Experimental**

**Materials/Characterisation**

Silver diethyldithiocarbamate (99%), tetramethylammonium hydroxide solution (97%, 25% weight in H_2_O), chloroform (>99.8%), ethanol (99.8%), dihydrolipoic acid (97%), 1-dodecanethiol (DDT, >98%), oleylamine (70%), hydrogen tetrachloroaurate (III) trihydrate (99.9%), IR-1061(80%), Amicon® Ultra-4 centrifugal filters (30 k) were purchased from Sigma-Aldrich and were used without further purification. A Millipore Milli-Q system (18 MΩ cm, 25°C) was used for purification of water before each use.

Absorption spectra were measured using a PerkinElmer Lambda 365 UV-Vis spectrometer (200 – 1100 nm) with the use of 10 mm path length quartz cuvettes. A PerkinElmer Lambda 750S UV-Vis-NIR spectrometer (200 – 2500 nm) with an integrating sphere setup was used carry out transmission measurements. The obtained data was analysed through Tauc plots; bandgap energies of the QDs were measured by fitting a tangent to the tail of the peak between 3 – 4 eV and taking the value of the x-intercept.

A Hettich Rotina 380R was used for centrifugation at 3600 rpm/2521 rcf.

An Edinburgh Instruments FS5 spectrophotometer was used to record all fluorescence measurements with quartz cuvettes of 10 mm path length. Emission spectra were measured in the visible-NIR I region with the use of a PMT-980 detector (300 – 950 nm) and in the NIR-II region with the use of a TE cooled Hamamatsu H10330C-75 NIR-PMT detector (950 – 1600 nm). Both detectors possess a reduced sensitivity between 920 – 1000 nm, hence emission signal was too low for reliable measurement in this region. Photoluminescent quantum yield (PLQY) was calculated using the absolute method by comparing integrated fluorescence intensity of the blank (solvent) to the quantum dots (QDs) dispersed in a solvent, with the use of the calculation, $\Phi=\frac{N_{Emitted photons}}{N_{Absorbed photons}}$. using an integrating sphere setup. PLQY was calculated for NIR-II emitting Ag_2_S QDs using the relative method, through comparison of serially diluted samples of Ag_2_S QDs and standard dye IR-1061 (Φ = 1.7%). Integrated fluorescence intensities and absorbances of samples were measured and PLQY was calculated using the equation, $\Phi_{U}= \Phi_{R} . \frac{m_{U}{}^{2}}{m_{R}{}^{2}} . \frac{\eta_{U}{}^{2}}{\eta_{R}{}^{2}}$. A picosecond pulsed diode laser (TCSPC) (Edinburgh Instruments EPL-405) with an excitation of 405 nm was used to measure fluorescence lifetime and the data was analysed using a tri-exponential fit.

Malvern Zetasizer Nano ZS was used to measure the hydrodynamic diameters and zeta potentials of prepared QDs with the use of quartz cuvettes of 10 mm path length and DTS1070 cells respectively.

An IVIS Lumina III was used to measure the NIR-I florescence phantoms using an emission filter at 840 nm, with an excitation wavelength of 410 nm. NIR images were measured using 1s exposure time at high lamp intensity, using medium image binning settings. ROIs with an identical shape/size were selected on the image around the liquid sample in Eppendorf tubes to measure counts in radiance units (p s^-1^cm^-^² sr^-1^); the mean counts and standard deviations generated from these ROIs were plotted.

The crystallographic state and phase formation of the AgAuS quantum dots (QDs) were examined using a X’Pert PRO diffractometer (PANalytical), operating at 45 kV and 40 mA, and utilising Cu Kα radiation (λ = 1.541874 Å). The measurements were conducted using Bragg-Brentano geometry over a 2θ scanning range of 25–65° at a scanning speed of 0.006° s^−1^. The obtained XRD patterns were matched against the Crystallography Open Database (COD) using the High Score software package (PANalytical).

X-ray photoelectron spectroscopy (XPS) measurements for the AgAuS quantum dots (QDs) were conducted using an ESCALAB 250 Xi system (Thermo Fisher Scientific, England) with monochromatic Al Kα radiation (1486.6 eV). The energy resolution (ΔE) was defined as the full width at half maximum (FWHM) of the Ag 3d5/2 peak after removal of a linearly interpolated background, with ΔE determined to be approximately 0.45 eV.

Scanning Transmission Electron Microscopy (STEM) and energy-dispersive X-ray spectroscopy (EDX) studies were performed using a double-corrected FEI Titan G3 Cubed Themis equipped with a Super-X EDX System, operating at 200 kV. For these studies, fresh samples were prepared by firstly dispersing the powder in chloroform and then drop-casting this on a holey carbon Ni grid.

Thermogravimetric analysis (TGA) of the samples was performed using a Mettler Toledo TGA/DSC 1/1100 SF STARe system from 25 to 900 °C. From room temperature, the samples were heated up to 120 °C at a heating rate of 10 °C/min, maintained at 120 °C for 10 min and then heated up to 800 °C at 10 °C/min, all under an argon flow rate of 30 mL/min. Approximately 10 mg sample was used for the analysis.

The mass of an average AgAuS QD was calculated by finding the number of unit cells in an average QD to obtain the total number of Ag, Au and S atoms to account for inorganic mass. The proportion of organic mass in surface ligands was determined through TGA. The sum of these masses gave an average QD molecular weight of approximately 888 kDa to enable the conversion of mg/mL to mM.

***Synthesis of Ag_2_S QDs***

To a three-necked flask, silver (I) diethyldithiocarbamate (54 mg, 0.21 mmol) was added and dried under vacuum for 5 min. 1-dodecanethiol (DDT) (25 mL) was added and the mixture was degassed under vacuum at room temperature (3 x 15 min), cycling between argon before each degassing period. The temperature of the reaction was increased to 210°C at a rate of 12 °C/min and the cores were grown for 45 min at this temperature. The solution of core Ag_2_S QDs was then allowed to cool until 100°C, at which point ethanol (100 mL) was quickly added to precipitate the QDs. The mixture was centrifuged (3600 rpm, 2521 rcf, 5 min) and the supernatant was discarded to leave a black pellet of DDT-coated Ag_2_S QDs. The pellet was resuspended in chloroform (40 mL) and stored in the dark at 4°C. (d = 6.38 ± 0.52 nm, λ_ex_ = 395 nm, λ_em_ = 1180 nm).

***Synthesis of Au-alloyed Ag_2_S QDs***

Half of the prepared DDT-coated Ag_2_S QDs in chloroform (20 mL) were added to a flask and a stock solution of hydrogen tetrachloroaurate (III) trihydrate in oleylamine/chloroform 1:6 added was varied volumetrically to achieve different ratios of Au:Ag. For the synthesis of AgAuS QDs, a solution of hydrogen tetrachloroaurate (III) trihydrate in oleylamine/chloroform 1:6 (40 mg/mL, 1 mL) was prepared. This solution was added quickly to the Ag_2_S QDs in chloroform with vigorous stirring and allowed to stir overnight at room temperature. The reaction was quenched by precipitation of AgAuS QDs with ethanol (60 mL), and centrifuged (3600 rpm, 2521 rcf, 5 min). The supernatant was discarded and the brown pellet of DDT-coated AgAuS QDs was resuspended in chloroform (20 mL) and stored in the dark at 4°C. (d = 5.76 ± 0.61 nm, λ_ex_ = 395 nm, λ_em_ = 795 nm).

***Aqueous phase transfer of AgAuS QDs***

DHLA (300 mg, 1.44 mmol) was dissolved in water (4 mL) and adjusted to pH 7.6 by addition of TMAOH. The DHLA solution was added to AgAuS QDs in chloroform (4 mL) in a vial, and the biphasic mixture was stirred vigorously at room temperature. The reaction was left for 1 h, until the AgAuS QDs had transferred completely from the lower chloroform layer to the upper aqueous layer. The aqueous layer was extracted and diluted with water (5 mL). The aqueous suspension containing LA-coated AgAuS QDs were transferred to 30 kDa centrifugal filters and washed repeatedly with water upon centrifugation to remove excess ligand (3600 rpm, 2521 rcf, 5 min). The LA-coated QDs were stored in a final volume of 1 mL, at 4°C in the dark. (λ_ex_ = 400 nm, λ_em_ = 845 nm).


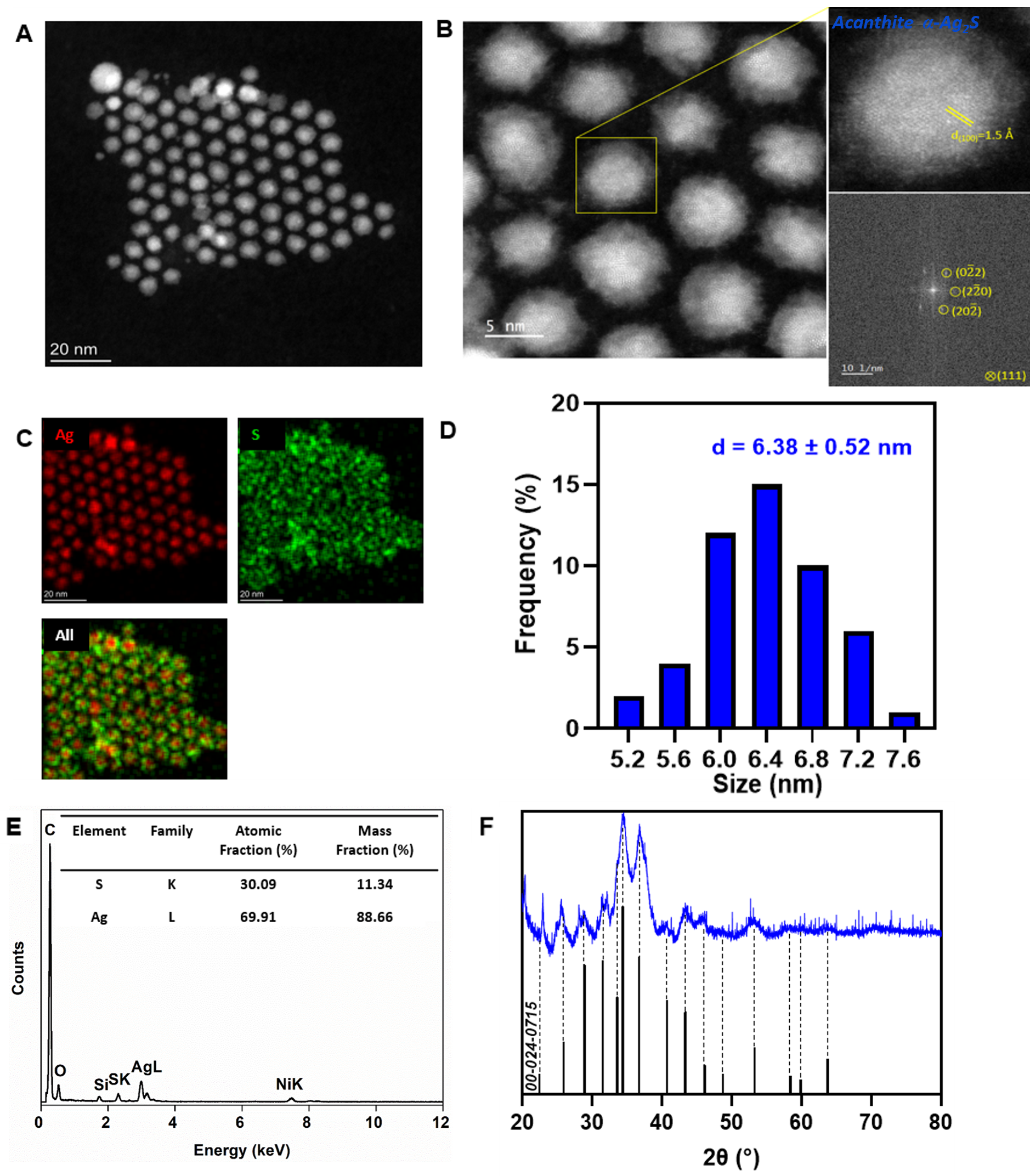


**Figure S1.** **(A)** HAADF-STEM of Ag_2_S QDs. **(B)** Corresponding zoomed HAADF-STEM and FFT inset to show the crystal structure of QDs, d-spacing and hkl assignment. **(C)** HAADF-STEM images of QDs with Ag and S content incorporated, coloured in red and green respectively. **(D)** Particle size distribution of 150 QDs measured from HAADF-STEM images. **(E)** Corresponding EDX spectra of QDs, with atomic assignment, and tabulated data to show the relative ratio of Ag and S. **(F)** XRD spectra of QDs (blue) with reference material monoclinic Ag_2_S, JCPDS 00-024-0715.


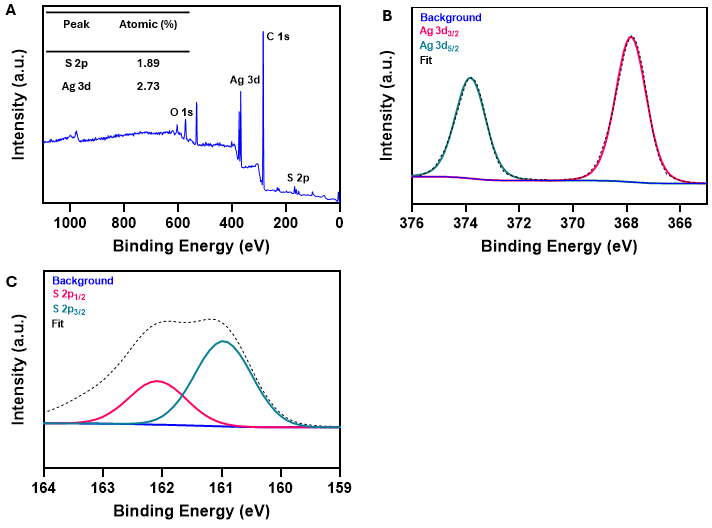


**Figure S2. (A)** Full XPS of Ag_2_S QDs, and tabulated data to show relative atomic contribution of silver and sulfur. Detailed comparisons of XPS between **(B)** Ag 3d and **(C)** S 2p.


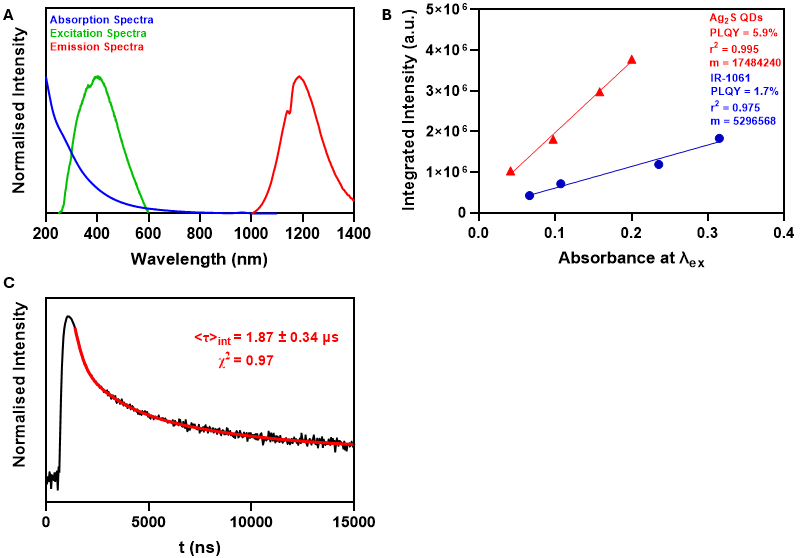


**Figure S3 (A)** Absorption, excitation (λ_em_ = 1180 nm) and emission (λ_ex_ = 400) spectra of Ag_2_S QDs. **(B)** Plot to calculate relative PLQY of Ag_2_S QDs through comparison with standard dye IR-1061 with known PLQY (Φ = 0.017). **(C)** Lifetime data measured using a TCSPC diode laser (λ_ex_ = 405 nm) and fitted with a tri-exponential decay. Tabulated fit data may be found at the end of the ESI. All measurements were made in chloroform at 25°C.


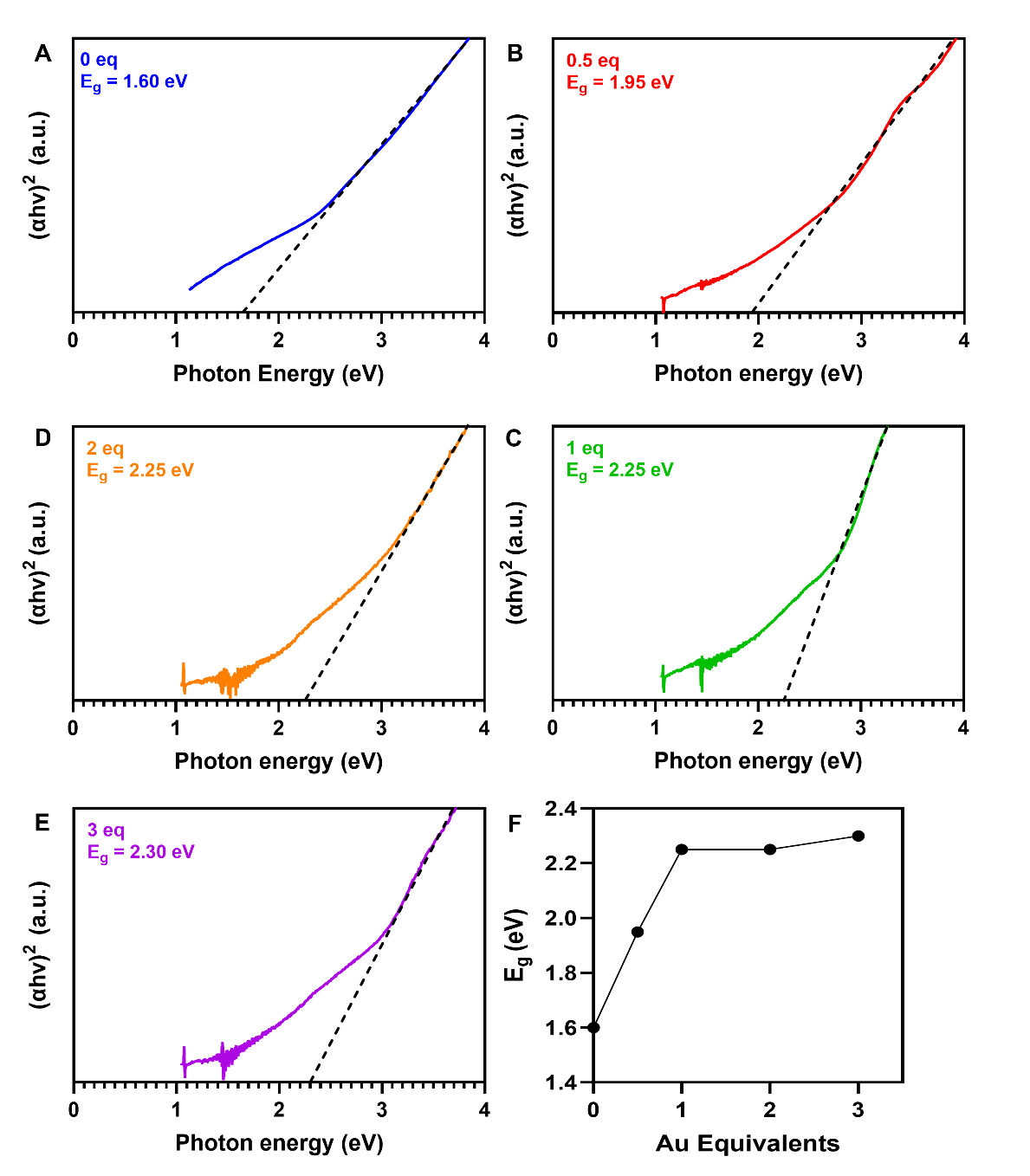


**Figure S4.** **(A)** Tauc plot of Ag_2_S QDs. Tauc plots of Au-alloyed Ag_2_S QDs using **(B)** 0.5, **(C)** 1, **(D)** 2 and **(E)** 3 eq. Au with respect to Ag. **(F)** Measured bandgap energies plotted against number of Au equivalents in the reaction. All absorption spectra were measured in chloroform at 25°C with the use of an integrating sphere setup.


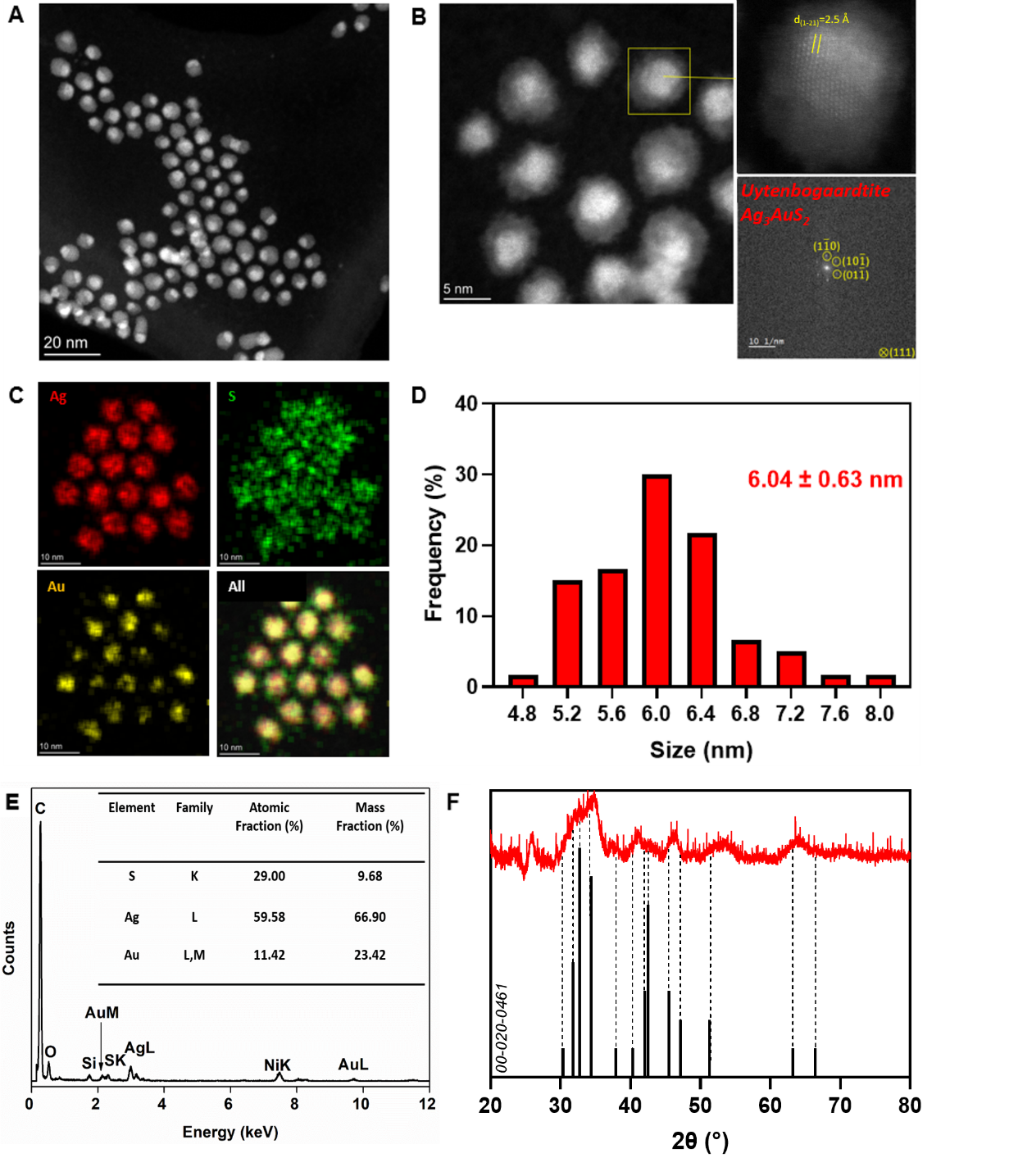


**Figure S5.** **(A)** HAADF-STEM of Ag_3_AuS_2_ QDs, alloyed using 0.5 eq. Au with respect to Ag. **(B)** Corresponding zoomed HAADF-STEM and FFT inset to show the crystal structure of QDs, d-spacing, and hkl assignment. **(C)** HAADF-STEM images of QDs with Ag, S, and Au content incorporated coloured in red, green, and yellow respectively. **(D)** Particle size distribution of 150 QDs measured from HAADF-STEM images. **(E)** Corresponding EDX spectra of QDs, with atomic assignment, and tabulated data to show the relative ratio of Ag, S, and Au. **(F)** XRD spectra of QDs (red) with reference material tetragonal Ag_3_AuS_2_, JCPDS 00-020-0461.


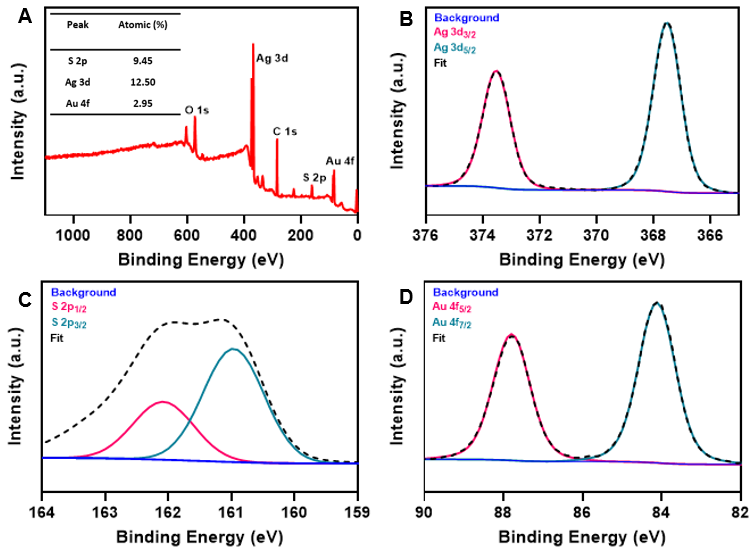


**Figure S6. (A)** Full XPS of Ag_3_AuS_2_ QDs, alloyed using 0.5 eq. Au with respect to Ag, and tabulated data to show relative atomic contribution of Ag, S and Au. Detailed comparisons of XPS between **(B)** Ag 3d, **(C)** S 2p and **(D)** Au 4f peaks.

**
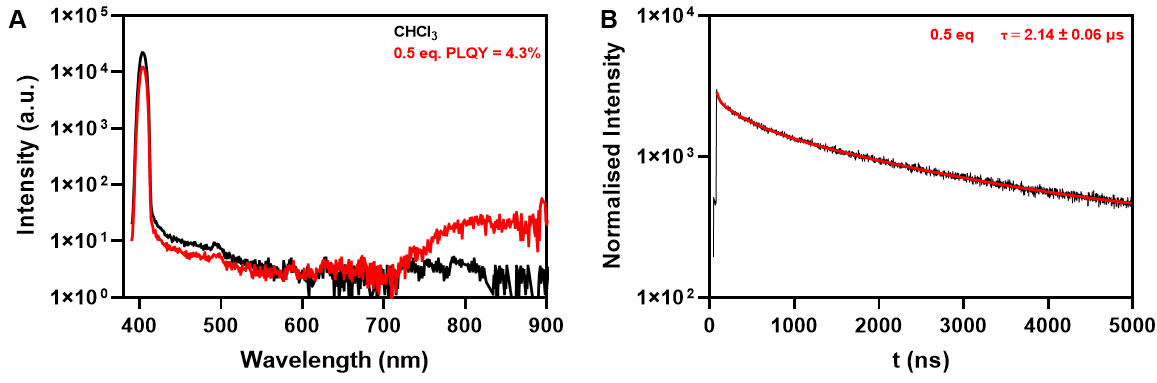
**

**Figure S7.** **(A)** Absolute PLQY (λ_ex_ = 395 nm) and **(B)** fluorescence lifetime measurement of Ag_3_AuS_2_ QDs, alloyed using 0.5 eq. of Au with respect to Ag. PLQY was measured using chloroform as the blank reference. All lifetime data was measured using a TCSPC diode laser (λ_ex_ = 405 nm) and fitted with a tri-exponential decay. Tabulated fit data may be found at the end of the ESI. All measurements were made in chloroform at 25°C.


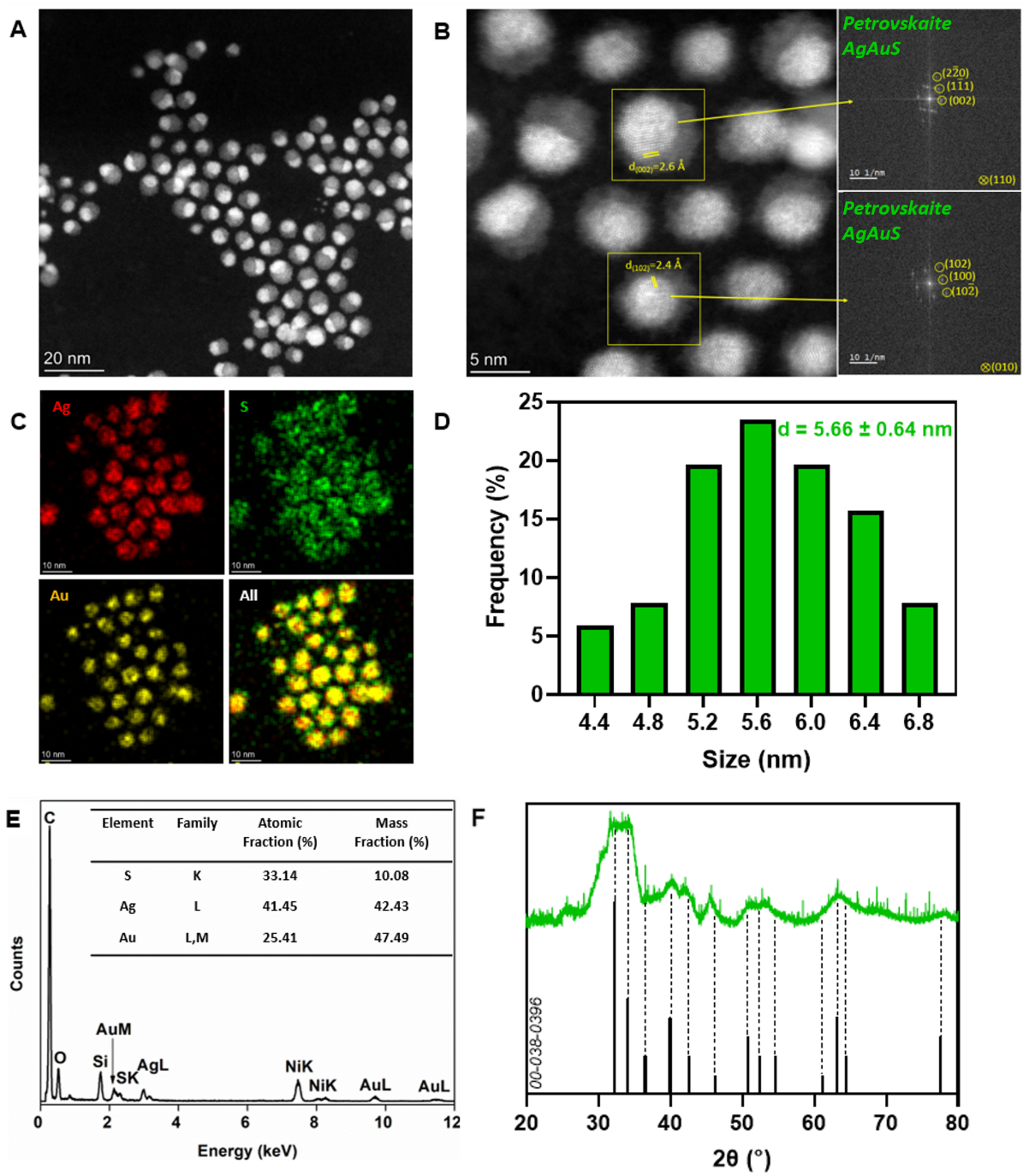


**Figure S8.** **(A)** HAADF-STEM of AgAuS QDs, alloyed using 1 eq. Au with respect to Ag. **(B)** Corresponding zoomed HAADF-STEM and FFT inset to show the crystal structure of QDs, d-spacing, and hkl assignment. **(C)** HAADF-STEM images of QDs with Ag, S, and Au content incorporated coloured in red, green, and yellow respectively. **(D)** Particle size distribution of 150 QDs measured from HAADF-STEM images. **(E)** Corresponding EDX spectra of QDs, with atomic assignment, and tabulated data to show the relative ratio of Ag, S, and Au. **(F)** XRD spectra of QDs (green) with reference material monoclinic AgAuS, JCPDS 00-038-0396.


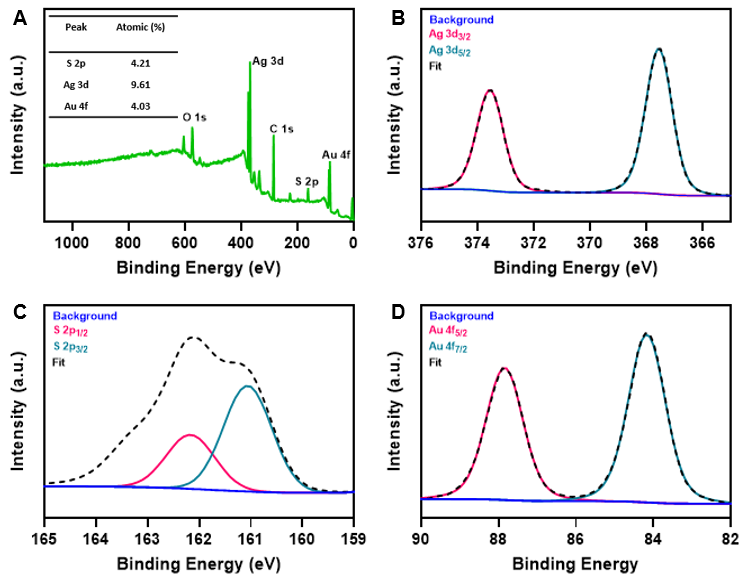


**Figure S9. (A)** Full XPS of AgAuS QDs, alloyed using 1 eq. Au with respect to Ag, and tabulated data to show relative atomic contribution of Ag, S and Au. Detailed comparisons of XPS between **(B)** Ag 3d, **(C)** S 2p and **(D)** Au 4f peaks.

**
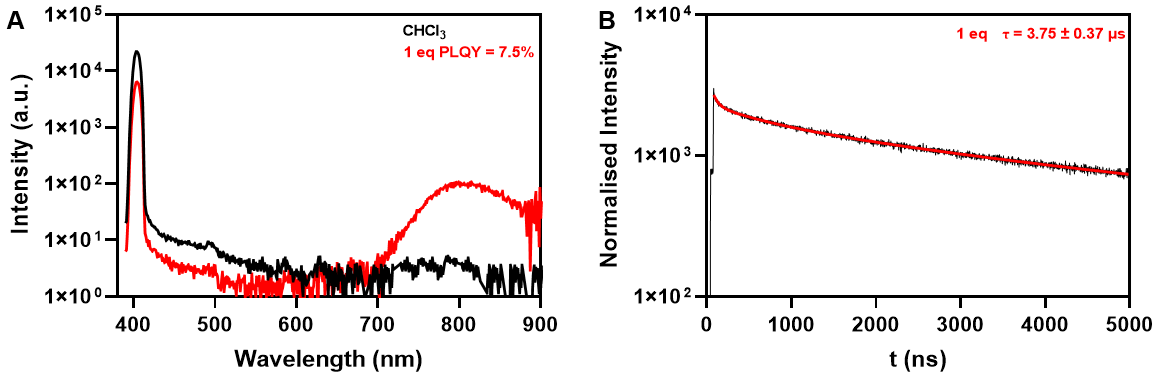
**

**Figure S10.** **(A)** Absolute PLQY (λ_ex_ = 395 nm) and **(B)** fluorescence lifetime measurement of AgAuS QDs, alloyed using 1 eq. of Au with respect to Ag. PLQY was measured using chloroform as the blank reference. All lifetime data was measured using a TCSPC diode laser (λ_ex_ = 405 nm) and fitted with a tri-exponential decay. Tabulated fit data may be found at the end of the ESI. All measurements were made in chloroform at 25°C.


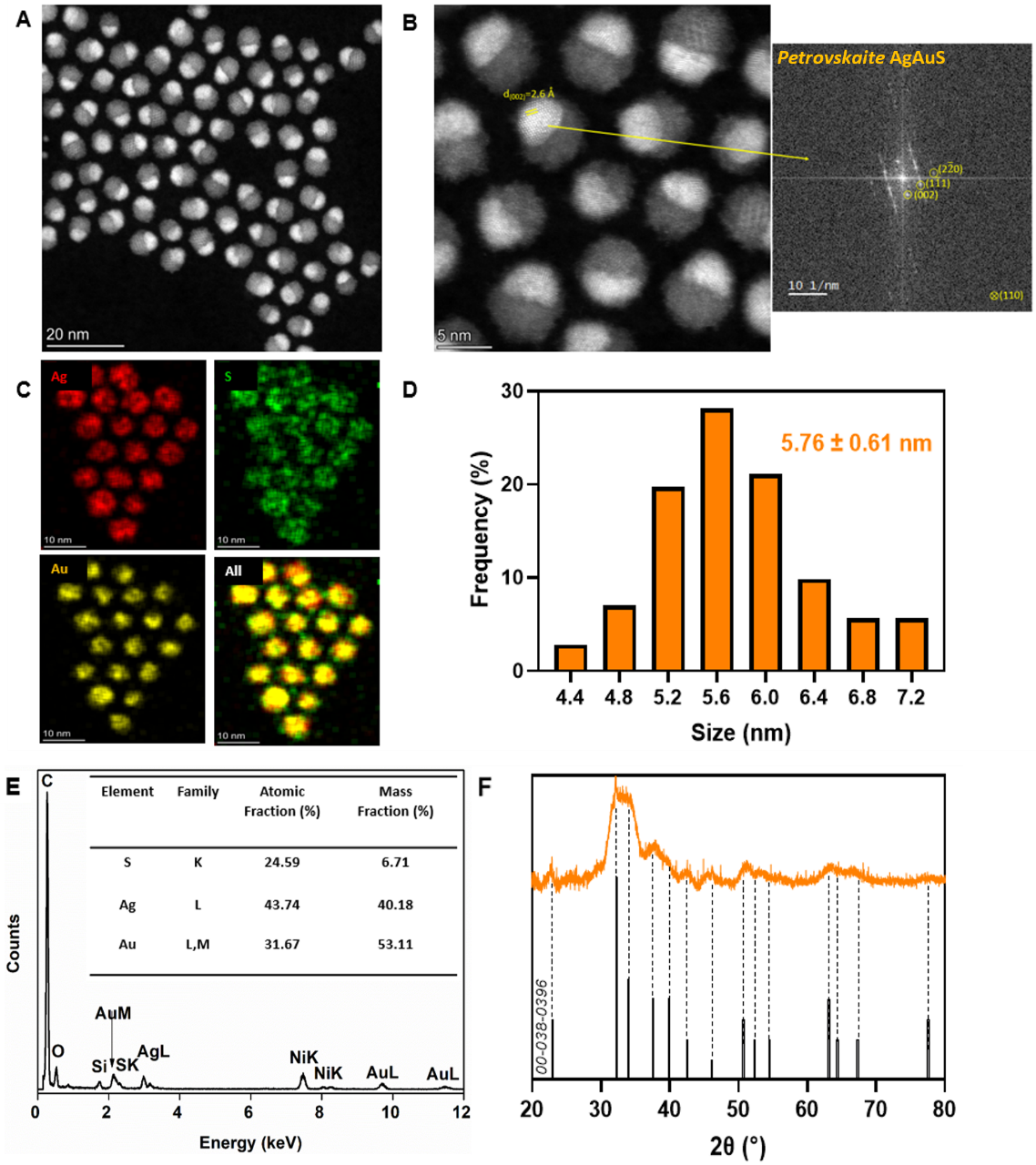


**Figure S11. (A)** HAADF-STEM of AgAuS QDs, alloyed using 2 eq. Au with respect to Ag. **(B)** Corresponding zoomed HAADF-STEM and FFT inset to show crystal structure of QDs, d-spacing, and hkl assignment. **(C)** HAADF-STEM images of QDs with Ag, S, and Au content incorporated coloured in red, green, and yellow respectively. **(D)** Particle size distribution of 150 QDs measured from HAADF-STEM images. **(E)** Corresponding EDX spectra of QDs, with atomic assignment, and tabulated data to show relative ratio of Ag, S, and Au. **(F)** XRD spectra of QDs (orange) with reference material monoclinic AgAuS, JCPDS 00-038-0396.


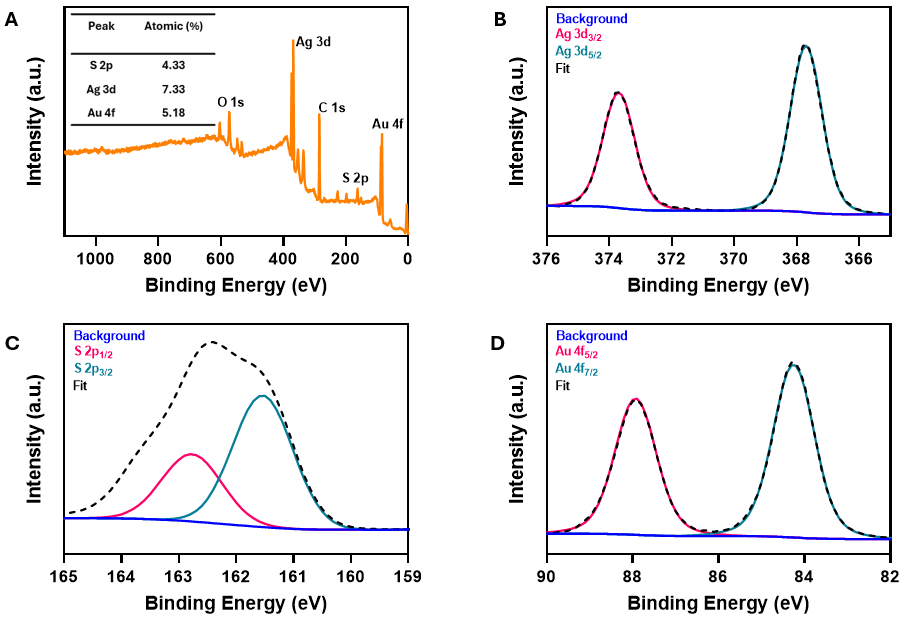


**Figure S12. (A)** Full XPS of AgAuS QDs, alloyed using 2 eq. Au with respect to Ag, and tabulated data to show relative atomic contribution of Ag, S and Au. Detailed comparisons of XPS between **(B)** Ag 3d, **(C)** S 2p and **(D)** Au 4f peaks.


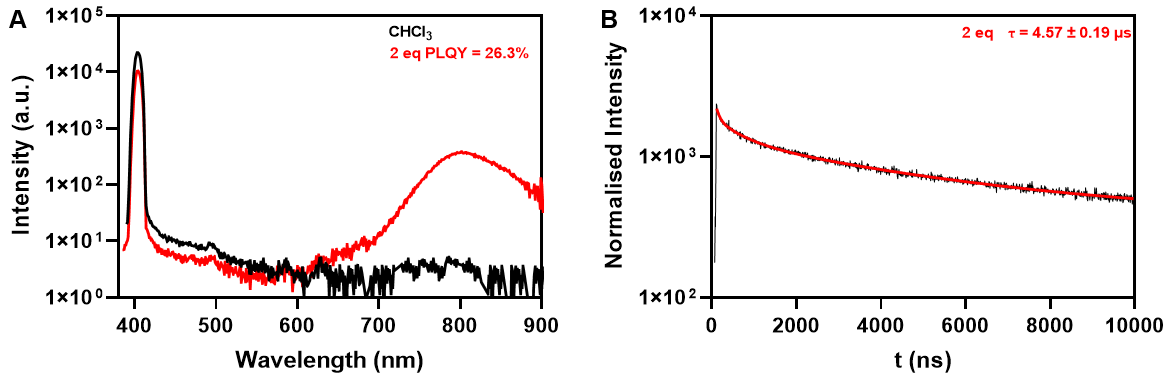


**Figure S13.** **(A)** Absolute PLQY (λ_ex_ = 395 nm) and **(B)** fluorescence lifetime measurement of AgAuS QDs, alloyed using 2 eq. of Au with respect to Ag. PLQY was measured using chloroform as the blank reference. All lifetime data was measured using a TCSPC diode laser (λ_ex_ = 405 nm) and fitted with a tri-exponential decay. Tabulated fit data may be found at the end of the ESI. All measurements were made in chloroform at 25°C.


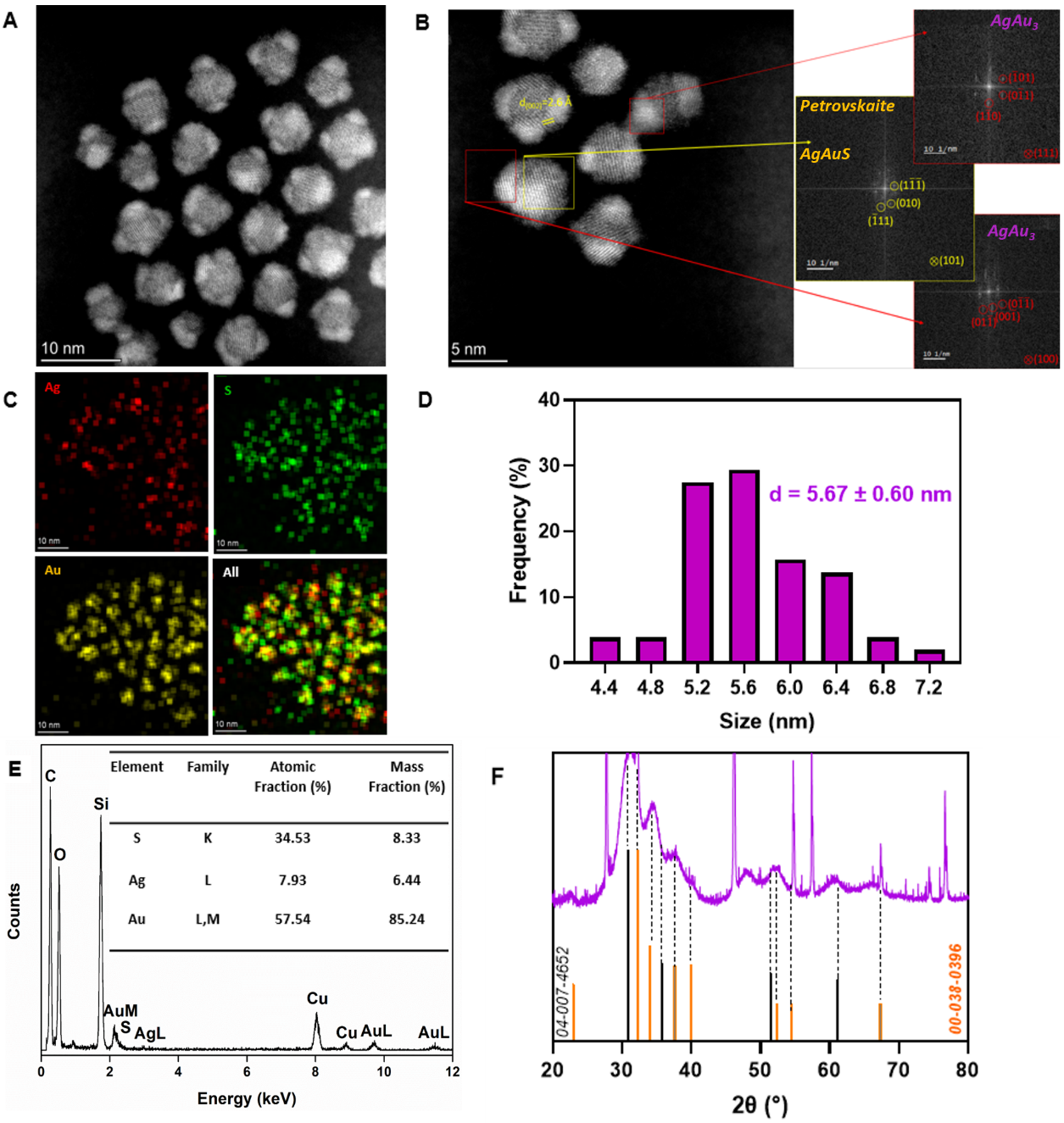


**Figure S14.** **(A)** HAADF-STEM of hybrid AgAu_3_-AgAuS QDs, alloyed using 3 eq. Au with respect to Ag. **(B)** Corresponding zoomed HAADF-STEM and FFT inset to show crystal structure of QDs, d-spacing, and hkl assignment. **(C)** HAADF-STEM images of QDs with Ag, S, and Au content incorporated coloured in red, green, and yellow respectively. **(D)** Particle size distribution of 150 QDs measured from HAADF-STEM images. **(E)** Corresponding EDX spectra of QDs, with atomic assignment, and tabulated data to show relative ratio of Ag, S, and Au. **(F)** XRD spectra of QDs (purple) with reference materials monoclinic AgAuS, JCPDS 00-038-0396 (orange) and cubic Au_2_S, 04-007-4652 (black).


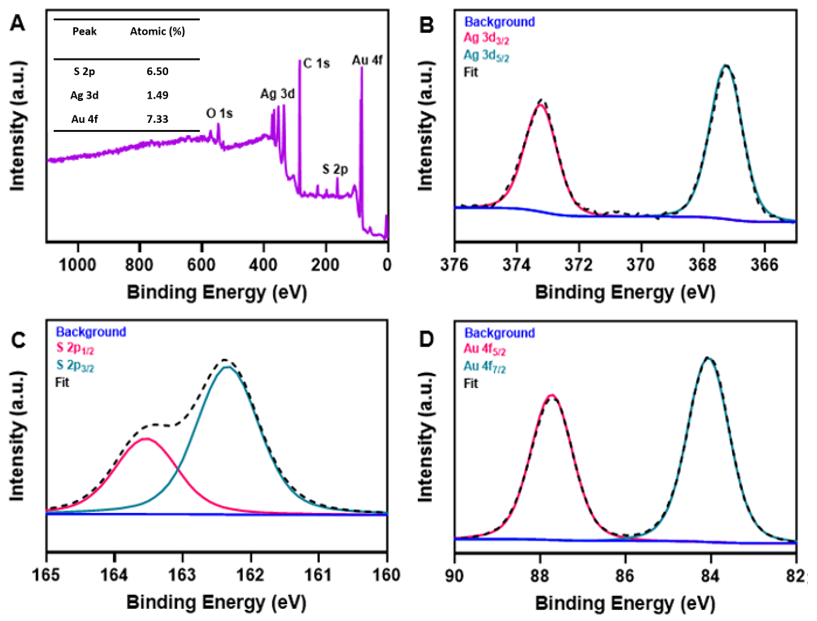


**Figure S15. (A)** Full XPS of hybrid AgAu_3_-AgAuS QDs, alloyed using 3 eq. Au with respect to Ag, and tabulated data to show relative atomic contribution of Ag, S and Au. Detailed comparisons of XPS between **(B)** Ag 3d, **(C)** S 2p and **(D)** Au 4f peaks.


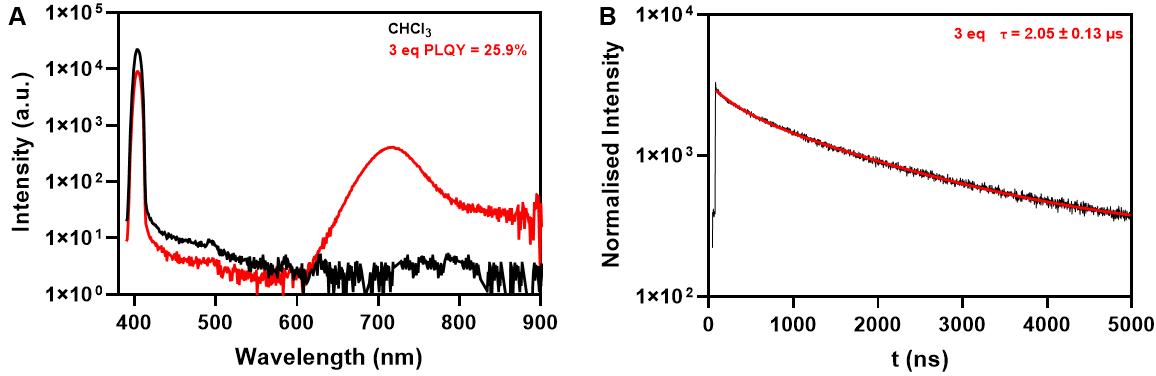


**Figure S16.** **(A)** Absolute PLQY (λ_ex_ = 395 nm) and **(B)** fluorescence lifetime measurement of AgAu_3_-AgAuS QDs, alloyed using 3 eq. of Au with respect to Ag. PLQY was measured using chloroform as the blank reference. All lifetime data was measured using a TCSPC diode laser (λ_ex_ = 405 nm) and fitted with a tri-exponential decay. Tabulated fit data may be found at the end of the ESI. All measurements were made in chloroform at 25°C.


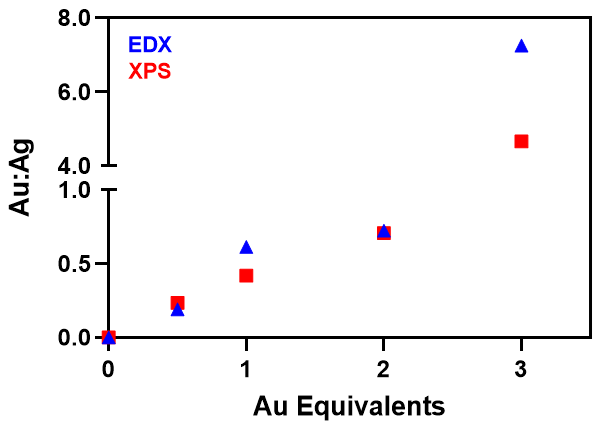


**Figure S17.** Ag:Au ratio incorporated in the QDs upon alloying with varying Au equivalents, measured through atomic contribution of Ag and Au through EDX and XPS measurements. Graph was plotted with a break in the y-axis between the data obtained for 2 and 3 eq. Au.


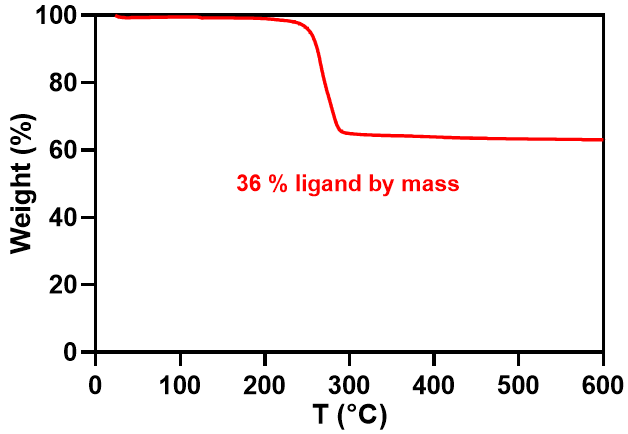


**Figure S18.** TGA data of phase transferred AgAuS QDs, for calculation of the proportion of ligands (DHLA) by mass that were bound to the QDs.


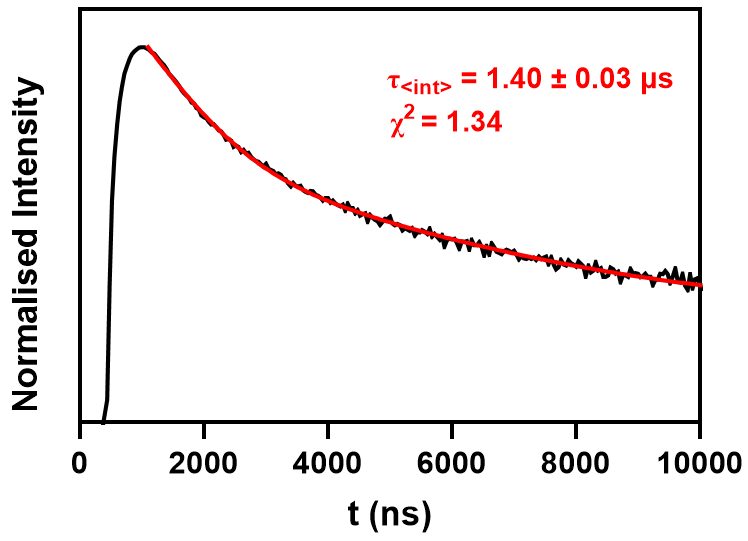


**Figure S19.** Fluorescence lifetime of phase transferred AgAuS QDs. Lifetime data was measured using a TCSPC diode laser (λ_ex_ = 405 nm) and fitted with a tri-exponential decay. Tabulated fit data may be found at the end of the ESI. Measurement was made in water at 25°C.


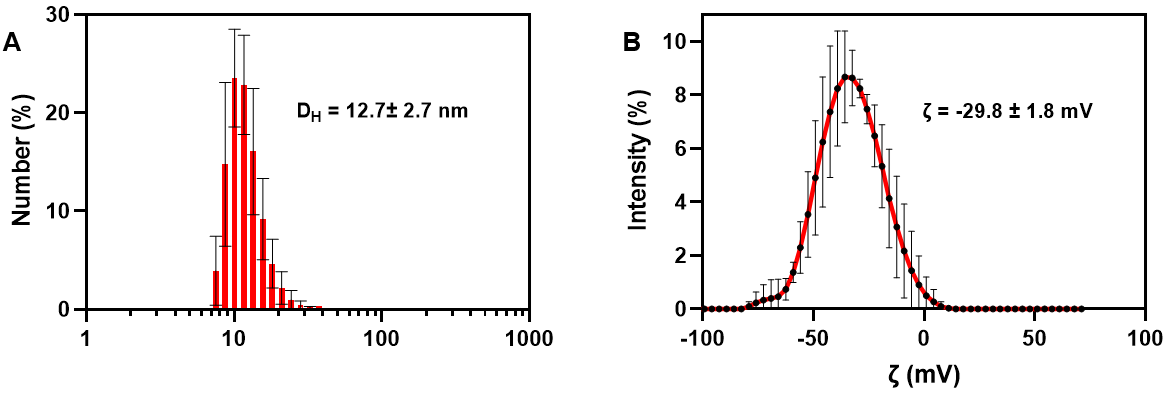


**Figure S20.**  **(A)** Hydrodynamic diameter (D_H_) and **(B)** zeta-potential distributions of phase transferred AgAuS QDs. All measurements were made in water at 25°C.


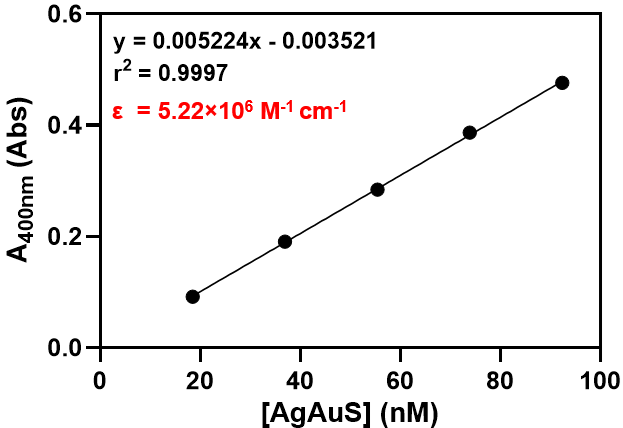


**Figure S21.** Calibration curve of phase transferred AgAuS QDs, for calculation of molar absorptivity at 400 nm. All absorbance measurements were made in water at 25°C.


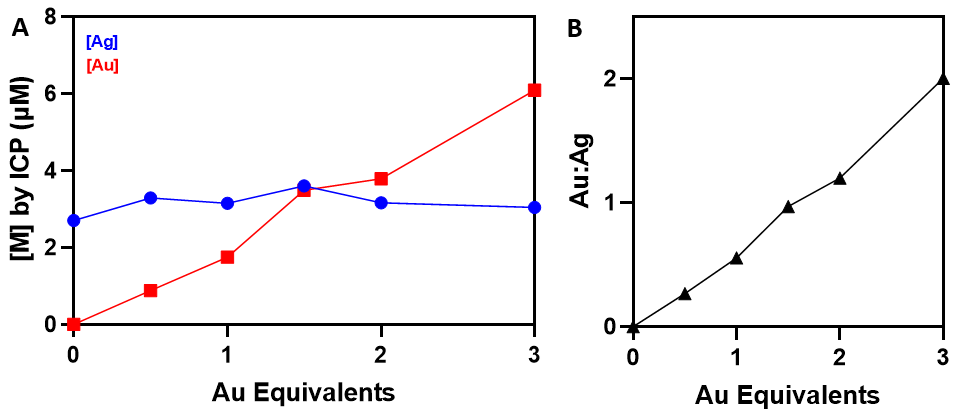


**Figure S21.** Relative concentrations of silver/gold measured through ICP-OES against equivalents of gold in the reaction with Ag_2_S-DDT QDs (A). Gold to silver ratio plotted from ICP-OES measurements against equivalents of gold in the reaction (B).


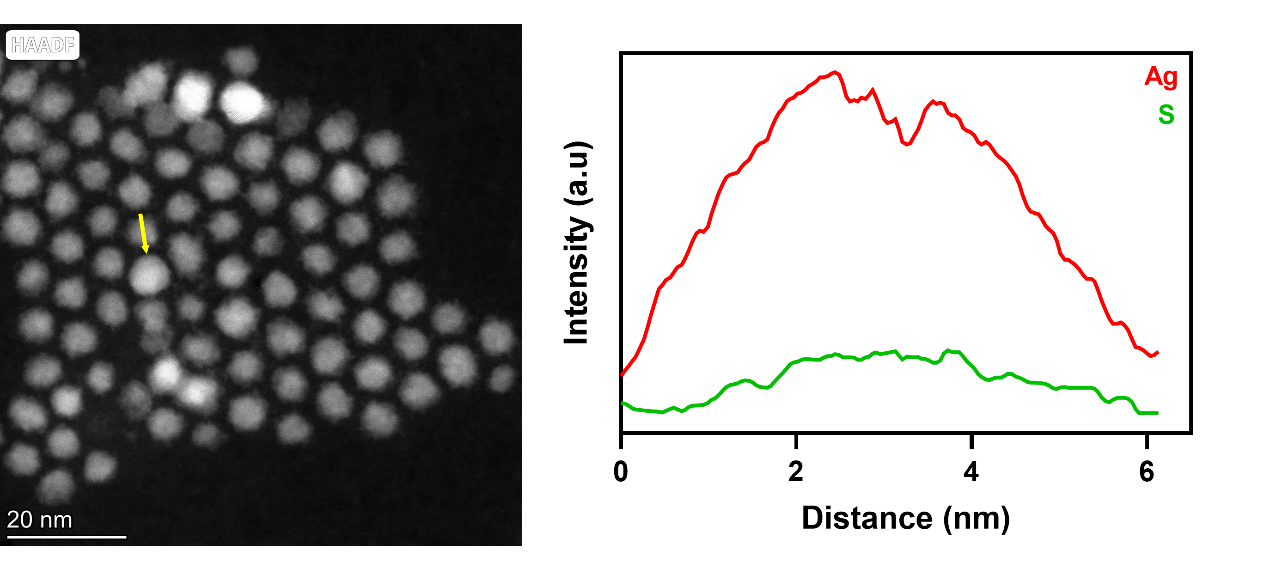


**Figure S22.** HAADF-STEM of Ag_2_S QDs (left) with corresponding EDX elemental line map of single measured nanoparticle (right).


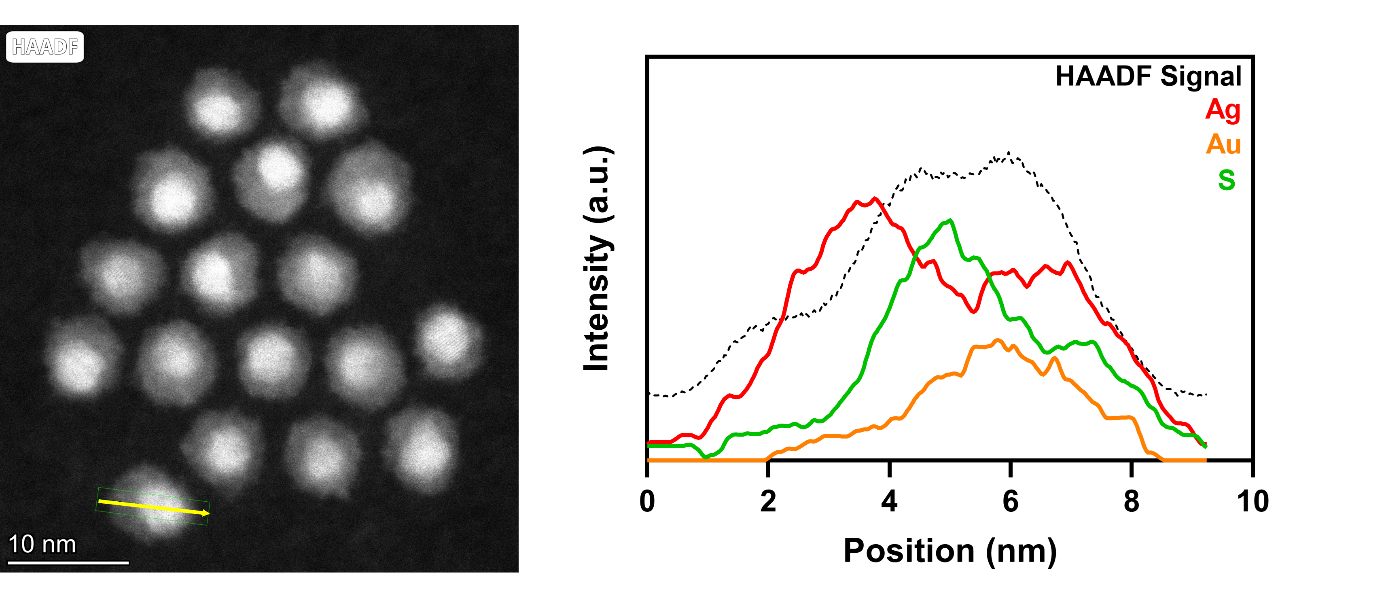


**Figure S23.** HAADF-STEM of Ag_3_AuS_2_ QDs, alloyed using 0.5 eq. Au with respect to Ag (left) with corresponding EDX elemental line map of single measured nanoparticle (right).


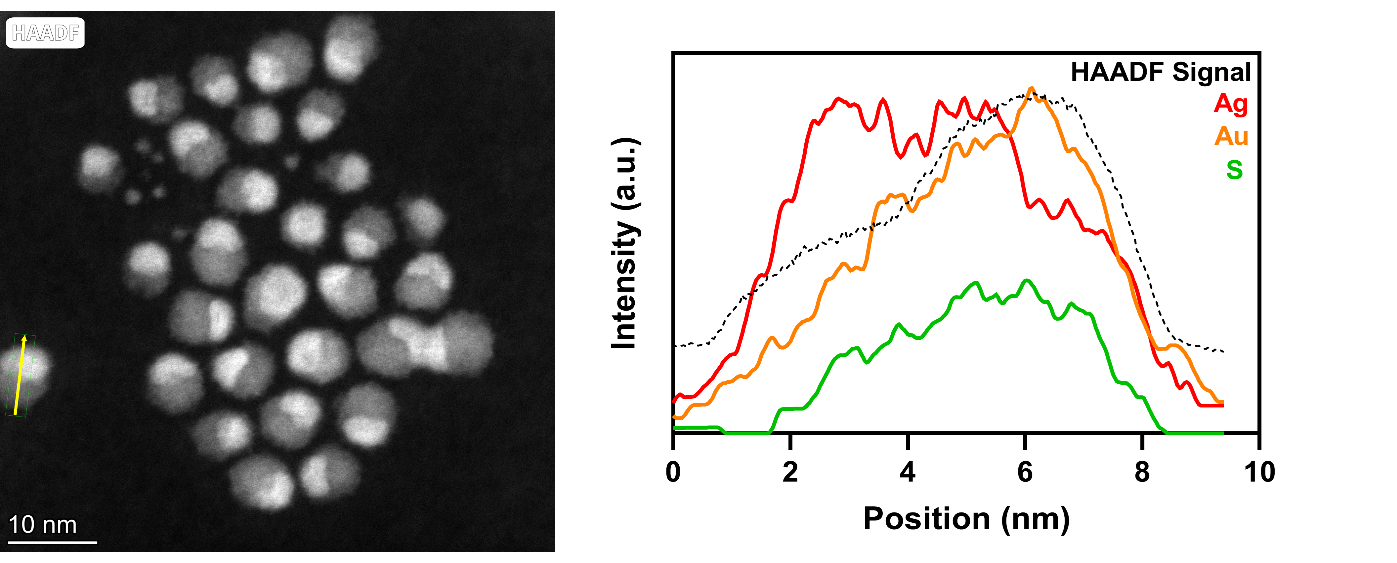


**Figure S24.** HAADF-STEM of AgAuS QDs, alloyed using 1 eq. Au with respect to Ag (left) with corresponding EDX elemental line map of single measured nanoparticle (right).


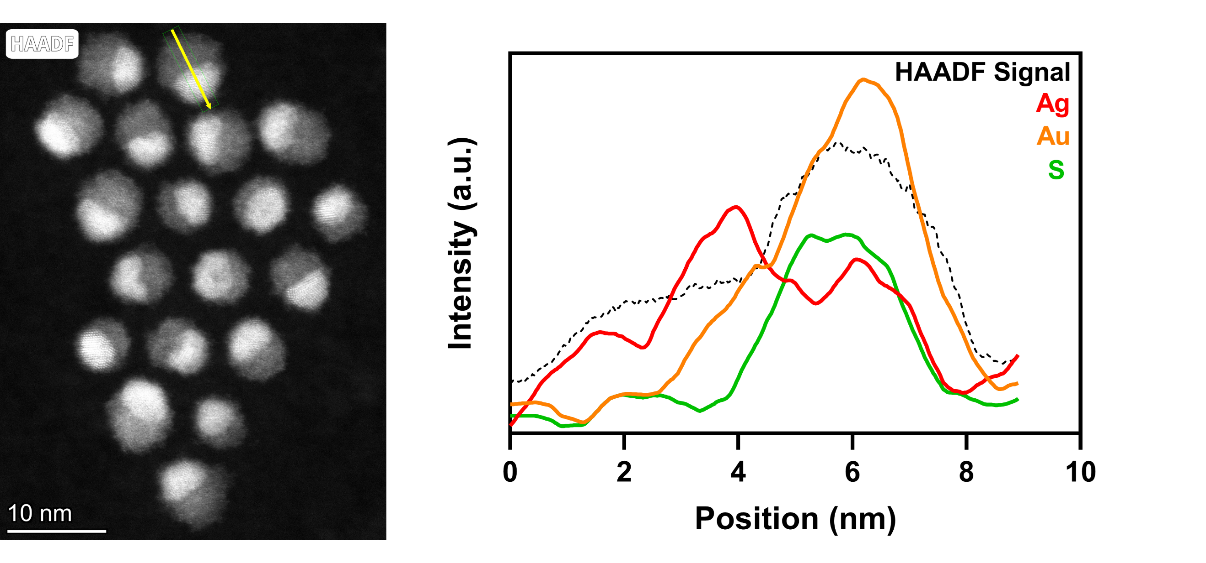


**Figure S25.** HAADF-STEM of AgAuS QDs, alloyed using 2 eq. Au with respect to Ag (left) with corresponding EDX elemental line map of single measured nanoparticle (right).


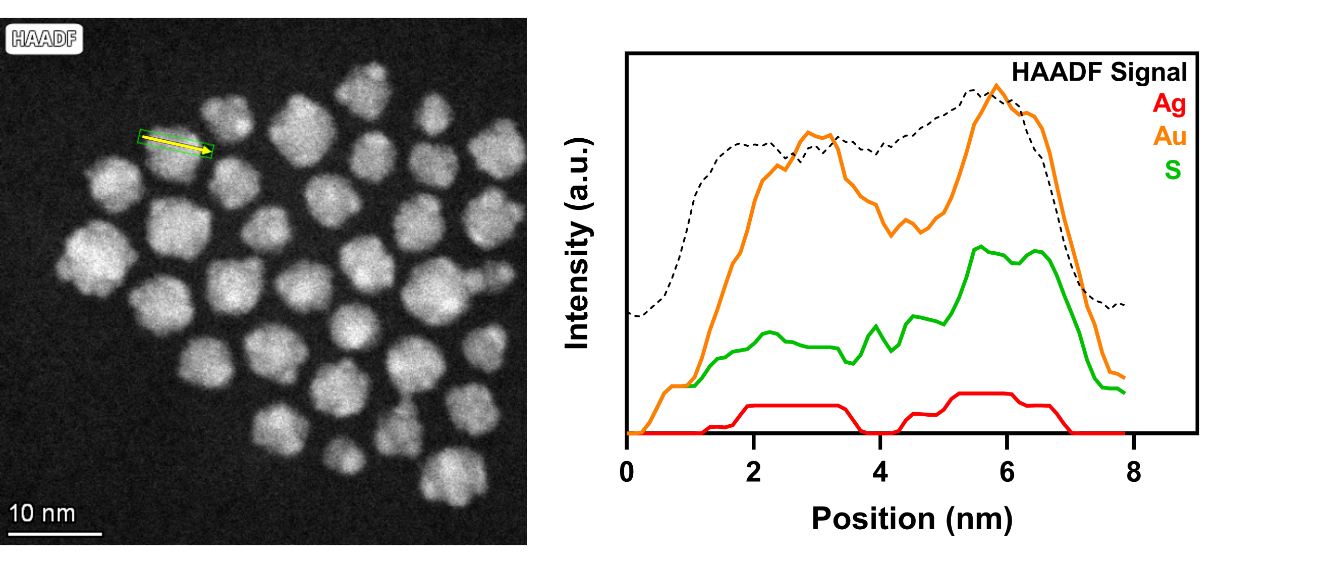


**Figure S26. (A)** HAADF-STEM of hybrid AgAu_3_-AgAuS, alloyed using 3 eq. Au with respect to Ag (left) with corresponding EDX elemental line map of single measured nanoparticle (right).

**Table S1.** Tri-exponential fluorescence lifetime fit result of Ag_2_S QDs, measured in chloroform at 25°C.

|  | Value (ns) | % |
| --- | --- | --- |
| τ_1_ | 222 | 31.6 |
| τ_2_ | 998 | 32.5 |
| τ_3_ | 4127 | 35.9 |
| < τ >_int_ | 1875 | - |
| Std. dev. | 336 | - |
| ꭓ2 | 0.97 | - |

**Table S2.** Tri-exponential fluorescence lifetime fit result of Ag_3_AuS_2_ QDs, alloyed with 0.5 eq. Au with respect to Ag, measured in chloroform at 25°C.

|  | Value (ns) | % |
| --- | --- | --- |
| τ_1_ | 28 | 0.3 |
| τ_2_ | 352 | 7.0 |
| τ_3_ | 2284 | 92.7 |
| < τ >_int_ | 2142 | - |
| Std. dev. | 55 | - |
| ꭓ2 | 1.04 | - |

**Table S3.** Tri-exponential fluorescence lifetime fit result of AgAuS QDs, alloyed with 1 eq. Au with respect to Ag, measured in chloroform at 25°C.

|  | Value (ns) | % |
| --- | --- | --- |
| τ_1_ | 58 | 0.4 |
| τ_2_ | 594 | 4.3 |
| τ_3_ | 4025 | 95.3 |
| < τ >_int_ | 3862 | - |
| Std. dev. | 450 | - |
| ꭓ2 | 1.03 | - |

**Table S4.** Tri-exponential fluorescence lifetime fit result of AgAuS QDs, alloyed with 2 eq. Au with respect to Ag, measured in chloroform at 25°C.

|  | Value (ns) | % |
| --- | --- | --- |
| τ_1_ | 80 | 0.6 |
| τ_2_ | 628 | 6.5 |
| τ_3_ | 5082 | 92.9 |
| < τ >_int_ | 4762 | - |
| Std. dev. | 295 | - |
| ꭓ2 | 1.03 | - |

|  | Value (ns) | % |
| --- | --- | --- |
| τ_1_ | 64 | 0.2 |
| τ_2_ | 311 | 6.0 |
| τ_3_ | 1802 | 93.8 |
| < τ >_int_ | 1709 | - |
| Std. dev. | 23 | - |
| ꭓ2 | 1.03 | - |

**Table S5.** Tri-exponential fluorescence lifetime fit result of hybrid AgAu_3_-AgAuS QDs, alloyed with 3 eq. Au with respect to Ag, measured in chloroform at 25°C.

**Table S6.** Tri-exponential fluorescence lifetime fit result of phase transferred AgAuS QDs, measured in water at 25°C.

|  | Value (ns) | % |
| --- | --- | --- |
| τ_1_ | 34 | 0.2 |
| τ_2_ | 390 | 50.1 |
| τ_3_ | 2595 | 49.7 |
| < τ >_int_ | 1404 | - |
| Std. dev. | 30 | - |
| ꭓ2 | 1.34 | - |
